# Ablation of Mto1 in zebrafish exhibited hypertrophic cardiomyopathy manifested by mitochondrion RNA maturation deficiency

**DOI:** 10.1093/nar/gkab228

**Published:** 2021-04-09

**Authors:** Qinghai Zhang, Xiao He, Shihao Yao, Tianxiang Lin, Luwen Zhang, Danni Chen, Chao Chen, Qingxian Yang, Feng Li, Yi-Min Zhu, Min-Xin Guan

**Affiliations:** Division of Medical Genetics and Genomics, The Children's Hospital, Zhejiang University School of Medicine and National Clinical Research Center for Child Health, Hangzhou, Zhejiang 310058, China; Institute of Genetics, Zhejiang University School of Medicine, Hangzhou, Zhejiang 310058, China; Zhejiang Provincial Key Lab of Genetic and Developmental Disorder, Hangzhou, Zhejiang 310058, China; Key Laboratory of Reproductive Genetics, Ministry of Education of PRC, The Women's Hospital, Zhejiang University School of Medicine, Hangzhou, Zhejiang 310006, China; Institute of Genetics, Zhejiang University School of Medicine, Hangzhou, Zhejiang 310058, China; Institute of Genetics, Zhejiang University School of Medicine, Hangzhou, Zhejiang 310058, China; Institute of Genetics, Zhejiang University School of Medicine, Hangzhou, Zhejiang 310058, China; Institute of Genetics, Zhejiang University School of Medicine, Hangzhou, Zhejiang 310058, China; Institute of Genetics, Zhejiang University School of Medicine, Hangzhou, Zhejiang 310058, China; Institute of Genetics, Zhejiang University School of Medicine, Hangzhou, Zhejiang 310058, China; Institute of Genetics, Zhejiang University School of Medicine, Hangzhou, Zhejiang 310058, China; Institute of Genetics, Zhejiang University School of Medicine, Hangzhou, Zhejiang 310058, China; Key Laboratory of Reproductive Genetics, Ministry of Education of PRC, The Women's Hospital, Zhejiang University School of Medicine, Hangzhou, Zhejiang 310006, China; Division of Medical Genetics and Genomics, The Children's Hospital, Zhejiang University School of Medicine and National Clinical Research Center for Child Health, Hangzhou, Zhejiang 310058, China; Institute of Genetics, Zhejiang University School of Medicine, Hangzhou, Zhejiang 310058, China; Zhejiang Provincial Key Lab of Genetic and Developmental Disorder, Hangzhou, Zhejiang 310058, China; Key Laboratory of Reproductive Genetics, Ministry of Education of PRC, The Women's Hospital, Zhejiang University School of Medicine, Hangzhou, Zhejiang 310006, China; Joint Institute of Genetics and Genome Medicine between Zhejiang University and University of Toronto, Hangzhou, Zhejiang 310058, China

## Abstract

Deficient maturations of mitochondrial transcripts are linked to clinical abnormalities but their pathophysiology remains elusive. Previous investigations showed that pathogenic variants in *MTO1* for the biosynthesis of τm^5^U of tRNA^Glu^, tRNA^Gln^, tRNA^Lys^, tRNA^Trp^ and tRNA^Leu(UUR)^ were associated with hypertrophic cardiomyopathy (HCM). Using *mto1* knock-out(KO) zebrafish generated by CRISPR/Cas9 system, we demonstrated the pleiotropic effects of Mto1 deficiency on mitochondrial RNA maturations. The perturbed structure and stability of tRNAs caused by *mto1* deletion were evidenced by conformation changes and sensitivity to S1-mediated digestion of tRNA^Gln^, tRNA^Lys^, tRNA^Trp^ and tRNA^Leu(UUR)^. Notably, *mto1^KO^* zebrafish exhibited the global decreases in the aminoacylation of mitochondrial tRNAs with the taurine modification. Strikingly, ablated *mto1* mediated the expression of *MTPAP* and caused the altered polyadenylation of *cox1*, *cox3*, and *nd1* mRNAs. Immunoprecipitation assay indicated the interaction of MTO1 with MTPAP related to mRNA polyadenylation. These alterations impaired mitochondrial translation and reduced activities of oxidative phosphorylation complexes. These mitochondria dysfunctions caused heart development defects and hypertrophy of cardiomyocytes and myocardial fiber disarray in ventricles. These cardiac defects in the *mto1^KO^* zebrafish recapitulated the clinical phenotypes in HCM patients carrying the *MTO1* mutation(s). Our findings highlighted the critical role of *MTO1* in mitochondrial transcript maturation and their pathological consequences in hypertrophic cardiomyopathy.

## INTRODUCTION

The maturation of mitochondrial RNAs for translation is the multi-step processes involved in transcription, nucleolytic processing, posttranscriptional nucleotide modifications, aminoacylation of tRNA and polyadenylation of mRNA (1–6). Defects in the maturation of mitochondrial RNA transcripts have been linked to an array of human diseases, including hypertrophic cardiomyopathy and lactic acidosis (2,6). In vertebrate mitochondrion, 2 rRNAs, 22 tRNAs and 13 polypeptides for oxidative phosphorylation system (OXPHOS) are encoded by its own genomes (mtDNA) (7,8). These mtDNAs bidirectionally transcribe the large polycistronic precursors, catalyzed by own transcription machinery (9,10). After transcription, the polycistronic precursors are processed to release 2 rRNAs, 22 tRNAs and 13 mRNAs, mediated by RNase P and RNase Z, respectively (11–13). Most mRNAs are then subjected to polyadenylation on their 3′ ends, synthesized by mitochondrial poly(A) polymerase (MTPAP) (14–16). These tRNAs undergo CCA addition, posttranscriptional nucleotide modifications and aminoacylation (17–21). Of these modifications, the nucleotides at position 34 (wobble position of anticodon) of tRNA are more prone to be modified than those at other positions of tRNAs and impact the stabilization of anticodon structure, fidelity and efficiency of translation (22–25). The nucleotides at position 34 (U, C or G) of mammalian mitochondrial tRNAs carry the diverse species of modifications including 5-formylcytidine (f^5^C), 5-taurinomethyluridine (τm^5^U) and 5-taurinomethyl-2-thiouridine (τm^5^s^2^U) (26–29). In particular, the biosynthesis of τm^5^s^2^U modification was catalyzed by highly conserved tRNA modifying enzymes GTPBP3, MTO1 and MTU1/TRMU in human mitochondrion (30–34). In human mitochondrion, MTU1/TRMU is responsible for the 2-thio group of τm^5^s^2^U in tRNA^Glu^, tRNA^Gln^ and tRNA^Lys^ (30,35), while GTPBP3 and MTO1 are involved in the formation of the 5-taurinomethyl group of tRNA^Glu^, tRNA^Gln^, tRNA^Lys^, tRNA^Trp^ and tRNA^Leu(UUR)^ (27,36,37).

The deficient modifications at U34 of mitochondrial tRNAs have been linked to human diseases (2,6,27,28,35,37–40). The deficient τm^5^s^2^U modification caused by mutations in the *TRMU* and *GTPBP3* genes was responsible for mitochondrial dysfunctions leading to clinical phenotypes, including deafness, reversible infantile liver failure, hypertrophic cardiomyopathy and lactic acidosis (35,41,42). Especially, the clinical features of MTO1 deficiency included hypertrophic cardiomyopathy, lactic acidosis, and developmental delay (27,43–46). The primary defect of MTO1 deficiency is the deficient taurine modification of mitochondrial tRNA (27,36,43). The MTO1 deficiency-induced failures in tRNA metabolism led to the impairment of mitochondrial translation and subsequent the deficiencies of OXPHOS complexes I and IV (27,36,43,44,46). However, the lack of MTO1-deficiency animal model makes us difficult to elucidate the pathogenic mechanism underlying the MTO1-deficiency-manifesting hypertrophic cardiomyopathy. To investigate if defects in MTO1 cause the hypertrophic cardiomyopathy *in vivo*, we generated the *mto1* knock-out (KO) zebrafish produced by genome editing using the CRISPR/Cas9 system (47). First, we evaluated the effect of *mto1* deficiency on cardiac function including cardiac looping and the size of cardiomyocyte, as well as the ultrastructure of cardiac myofibrils. These *mto1*^ko^ zebrafish were further assessed for the *in vivo* effects of *mto1* deficiency on mitochondrial tRNA metabolism, mitochondrial translation and enzymatic activities of OXPHOS complexes. To investigate the molecular changes of *mto1*-deficiency, we performed mRNA-sequencing on whole hearts of *mto1*^−/−^ and WT zebrafish. The analysis of transcriptome of *mto1*^−/−^ and WT zebrafish hearts revealed the lower expression of genes involved in mitochondrial biogenesis including the *mtpap* encoding mitochondrial poly(A) polymerase (15,16). To examine if MTPAP directly interacts with MTO1, we performed the immunoprecipitation assay. Finally, we examine if the deletion of *mto1* affected the polyadenylation of mitochondrial mRNAs.

## MATERIALS AND METHODS

### Experimental fish and maintenance

AB wild-type strain and myocardium-specific transgenic *Tg* (*cmlc2*: *egfp*) zebrafish (*Danio rerio*) were used for this investigation. The animal protocols used in this investigation were approved by the Zhejiang University Institutional Animal Care and Use Committee. All fish were kept in recirculating water at 28°C and fed with commercial pellets at a daily ration of 0.7% of their body weight. Embryos were reared at 28.5°C according to standard protocols (48). Embryos were staged by hours post fertilization (hpf) and days post fertilization (dpf) (49).

### Generation of *Mto1* knock-out zebrafish by CRISPR/Cas9 system

The zCas9 expression plasmid pSP6–2sNLS-spCas9 was linearized by XbaI and used as a template for Cas9 mRNA *in vitro* synthesis with mMESSAGE mMACHINE mRNA transcription synthesis kits (Ambion). The sequence of sgRNAs was designed according to criteria as described previously (47). The gRNA transcription plasmid was pT7-gRNA. We used the CRISPR/Cas9 design tool (http://zifit.partners.org) to select specific targets to minimize off-target effects. Cas9-encoding mRNA (300 ng/μl) and gRNA (200 ng/μl) were co-injected into one-cell-stage wild-type embryos. Injected embryos were incubated at 28.5°C, and collected for making genomic DNA for genotyping at 50 hpf. Genomic DNA of the 50 hpf injected embryo was used as template to amplify *mto1* gene by using the following two primers: F1: TTTTGTCTTGTAGGGGCACT; R1: GTGGGCGGATTGA GTGAC. The fragments were cloned by using the TA Cloning Kit (TAKARA) and then were sequenced.

### Whole mount *in situ* hybridization

Probes were synthesized with digoxigenin (DIG)-labeled antisense RNA probes specific to zebrafish *mto1*, *mtpap* and *cmlc2* by using the primers depicted in Supplemental Table S1. WISH was carried out as detailed elsewhere (50–52). Zebrafish embryos from various age of post-fertilization were dechlorinated in 2 mg/ml pronase in E3 medium and fixed at 4°C in 4% paraformaldehyde in phosphate-buffered saline (PBS) overnight, then transferred to 100% methanol for storage at −20°C for at least 20 min before undergoing hybridization. After the hybridization procedure, embryos were washed extensively in PBS with 0.1% Tween 20, re-fixed in 4% paraformaldehyde, and then transferred to 70% glycerol. Stained embryos were visualized using stereoscopic microscopes (SMZ18, Nikon) (53).

### Histological studies

For hematoxylin and eosin (H&E) staining, zebrafish was anesthetized, their hearts were extracted and fixed in 10% formalin at 4°C overnight. Samples were then dehydrated, infiltrated, embedded in paraffin, sliced into 5 μm thick by pathologic microtome (RM2016, Leica). Tissue sections were then stained by H&E as described previously (54).

For wheat germ agglutinin (WGA) (Sigma) immunofluorescent staining, heart tissues were fixed in 4% paraformaldehyde at 4°C overnight, then washed with PBS and incubated in 30% sucrose solution at 4°C overnight. The samples were frozen in OCT compound at −80°C, and 10 μm cryo-sections were cut using a CM1950 cryostat (Leica). Frozen sections were washed in PBS for 10 min before incubated with FITC-conjugated WGA (1:50 in PBS, 1 mg/ml stock solution) for 1 h at room temperature. The nuclei were stained with DAPI (1:1000 in PBS, 10 μM stock solution, Sigma) for 10 min at room temperature (55,56). Fluorescence was visualized with a confocal microscope (OLYMPUS, FV1000).

### Transmission electron microscopy

The ultrastructure of adult zebrafish hearts at 6 month old was observed using transmission electron microscopy. Heart-tissues were fixed in 2.5% glutaraldehyde, embedded in Epon 812, and cut into 100-nm thick slices using UC7 ultramicrotome (Leica, Heerbrugg, Switzerland), then stained with uranyl acetate and lead citrate. Finally, the images of myocardial ultra-structure were captured by a Hitachi-7650 transmission electron microscope (Hitachi, Tokyo, Japan).

### Mitochondrial tRNA analysis

Total RNAs were isolated from fish using Totally RNA™ Kit (Ambion, Inc). The presence of thiouridine modification in the tRNAs was verified by the retardation of electrophoretic mobility in a polyacrylamide gel that contains 0.05 mg/ml (*N*-acryloylaminophenyl) mercuric chloride (APM) (30,31,53). Total RNAs were separated by polyacrylamide gel electrophoresis and blotted onto positively charged membrane (Roche Applied Science). Each tRNA was hybridized with the specific DIG-oligodeoxynucleoside probes at the 3′ termini, as detailed elsewhere (56,57). Oligodeoxynucleosides used for DIG-labeled probes were zebrafish mitochondrial tRNA^Glu^, tRNA^Lys^ and tRNA^Ala^ as detailed elsewhere (Supplemental Table S2) (53,56). The oligodeoxynucleotides were generated by using DIG-oligonucleotide Tailing kit (Roche). APM gel electrophoresis, hybridization and quantification of 2-thiouridine modification in tRNAs were performed as detailed elsewhere (30,31,53). Hydrogen peroxide (H_2_O_2_) treatment was carried out as a nonthiolated control, as described elsewhere (58).

For tRNA Northern blot analysis, 5 μg of total RNAs were electrophoresed through a 10% polyacrylamide gel without (native gel) or with (denature gel) 8 M urea in Tris–borate–EDTA buffer (TBE) (after heating the sample at 65°C for 10 min), and then electroblotted onto a positively charged nylon membrane for the hybridization analysis with DIG-labeled oligodeoxynucleotide probes. Oligodeoxynucleoside probes for mitochondrial tRNA^Lys^, tRNA^Glu^, tRNA^Gln^, tRNA^Trp^, tRNA^Leu(UUR)^, tRNA^Ala^, tRNA^Met^ and 5S rRNA were described as previously (53,56). The hybridization and quantification of density in each band were performed as detailed previously (59,60).

For tRNA aminoacylation analysis, 5 μg of total RNA were electrophoresed at 4°C through an acid (pH 5.2) 10% polyacrylamide/8 M urea gel to separate the charged and uncharged tRNA as detailed elsewhere (59,61). The gels were electroblotted onto a positively charged nylon membrane (Roche) for the hybridization analysis with oligodeoxynucleotide probes specific tRNA^Lys^, tRNA^Gln^, tRNA^Leu(UUR)^, tRNA^Trp^, tRNA^Ala^ and tRNA^Met^, as detailed elsewhere (53,56). Quantification of density in each band was performed as detailed previously (59,61).

The S1 nuclease cleavage analysis was performed as detailed elsewhere (56,59). In brief, 2 μg of total RNAs were used for the cleavage reaction in the presence of 1 μg/μl total yeast tRNA and 1 U/μl S1 nuclease (Thermofisher) in the 5 μl reaction buffer containing 40 mM sodium acetate (pH 4.5), 300 mM NaCl and 2 mM ZnSO_4_. Reaction mixtures were incubated at 28°C for indicated times and quenched by adding 5 μl loading buffer. Samples were electrophoresed through a 10% denaturing polyacrylamide gel with 8 M urea and then electroblotted onto a positively charged nylon membrane for hybridization analysis with 3′end DIG-labeled oligodeoxynucleotide probes as described previously (56).

### Western blotting analysis

Western blotting analysis was carried out as detailed elsewhere (60,62). Fish were sacrificed after anesthesia and homogenized in RIPA reagent (Invitrogen) using a homogenizer. Twenty micrograms of total proteins were electrophoresed through 10% bis-Tris SDS-polyacrylamide gels and then transferred to a polyvinyl difluoride (PVDF) membrane. The antibodies used for this investigation were from Sigma [Mto1 (Sigma, HPA030232) and Gapdh (SAB2701826)], Abcam [Nd1 (ab74257), Sdha (ab151684), Atp5a (ab188107), Mtpap (ab154555) and Uqcrc2 (ab203832)] and Proteintech [Co2 (55070-1-AP), Atp8 (26723-1-AP), Ndufs1 (12444-1-AP), Cox5a (11448–1-AP), Atp5c (60284-1-Ig), Tfam (19998-1-AP), and Tufm (26730-1-AP) and Tom20 (1802-1-AP)]. Peroxidase AffiniPure goat anti-mouse IgG and goat anti-rabbit IgG (Jackson) were used as secondary antibodies and protein signals were detected using the ECL system (CWBIO). Quantification of density in each band was carried out as detailed previously (60).

### Blue native polyacrylamide gel electrophoresis analysis

Blue native polyacrylamide gel electrophoresis (BN-PAGE) was performed by isolating mitochondrial proteins from mutant and wild type zebrafish, as detailed previously (63,64). Samples containing 15 μg of proteins were separated on 3–11% Bis–Tris Native PAGE gel. The primary antibodies applied for this experiment were Ndufs1, Sdha, Uqcrc2, Cox5a and Atp5c. Peroxidase AffiniPure goat anti-mouse IgG and goat anti-rabbit IgG (Jackson) were used as secondary antibodies and protein signals were detected using the ECL system (CWBIO).

### Assays of activities of OXPHOS complexes

The enzymatic activities of complexes I, II, III, IV and V were assayed as detailed elsewhere (56,65). Briefly, the activity of complex I was determined through the oxidation of NADH with ubiquinone as the electron acceptor. Complex II was examined through the artificial electron acceptor DCPIP. The activity of complex III was measured through the reduction of cytochrome *c* (III) by using d-ubiquinol-2 as the electron donor. The complex IV was monitored through the oxidation of cytochrome *c* (II). The activity of complex V was explored through the NADH oxidation via conversion of phosphoenolpyruvate to lactate by two step reactions.

Enzyme histochemistry (EHC) analysis for succinate dehydrogenase (SDH) and cytochrome c oxidase (COX) in the frozen-sections were performed as detailed elsewhere (64,66). Briefly, freshly dissected heart tissues were embedded in OCT compound (Tissue-Tek), frozen on dry ice, and sectioned to 10 μm. For SDH assay, samples were incubated in 5 mM phosphate buffer, pH 7.6, containing 5 mM EDTA, 1 mM potassium cyanide (KCN), 0.2 mM phenazine methosulfate (PMS), 50 mM succinic acid, 1.5 mM nitro blue tetrazolium (NBT) at 37°C for 25 min. For COX assay, samples were incubated in 5 mM phosphate buffer, pH 7.4, containing 0.1% 3,3′-diaminobenzidine (DAB), 0.1% Cytochrome *c*, 0.02% catalase at 37°C for 60 min. For sequential COX/SDH assay, samples were incubated in the COX-solution as described above at 37°C for 20 min, and followed with SDH-solution as described above at 37°C for 20 min.

### RNA sequencing analysis

Total RNA was extracted from the hearts of *mto1*^−/−^ and WT zebrafish at 4-6 month old by using TRIzol Reagent (Takara) according to its protocol. RNA-seq libraries construction, and sequencing were performed by the Novogene Bioinformatics Institute (Beijing, China). Briefly, 1 μg RNA per sample was used as input materials to prepare the libraries following the standard procedures of NEBNext® UltraTM RNA Library Prep Kit for Illumina® (NEB, USA). Clustering of the index-coded samples was performed on a cBot Cluster Generation System using TruSeq PE Cluster Kit v3-cBot-HS (Illumia) according to the manufacturer's instructions. After cluster generation, the library preparations were sequenced on an Illumina Novaseq platform and 150 bp paired-end reads were generated. The filtered reads were aligned to the zebrafish reference genome (Ensembl release 97) by using TopHat (version 2.1.0). After the alignment analysis, the BAM files of each individual alignment were used to analyze genes differential expression by using Cufflinks (version 2.2.1). Genes with an adjusted *P*-value <0.01 and |log_2_ (fold change)| >1.58 were assigned as differentially expressed. The RNA-seq data have been deposited to NCBI database with the BioProject ID PRJNA 695802.

### Quantitative real-time PCR

Quantitative real-time PCR was performed using total RNAs extracted from *mto1*^−/−^ and WT zebrafish using Totally RNA™ Kit (Ambion) and reverse transcribed with the PrimeScript™ RT Reagent Kit (Takara). FastStart Universal SYBR Green Masters (Roche) were utilized and the 2^–ΔΔCt^ method was used to normalize the genes of interest (Supplementary Table S1) to the endogenous housekeeping gene *ef1α*.

### Immunoprecipitation (IP) assay

The cDNA of human *MTO1* was cloned into pcDNA3.1-Flag to construct expression vectors pcDNA3.1-MTO1-FLAG (C-terminal FLAG tag) using primers listed in Supplementary Table S1.

For transient expression of *MTO1*, HEK 293T cells (2.0 × 10^6^) were transfected with 12 μg of pcDNA3.1-MTO1-FLAG for 48 h using Hieff Trans liposomal transfection reagent following the manufacturer's protocol (Yeasen), as detailed elsewhere (67). The cells were harvested and suspended with 1 ml of lysis buffer [1% NP40; 50 mM Tris–HCl, pH7.6; 150 mM NaCl; 1× Protease Inhibitor Cocktail (Bimake)] and lysed on ice for 30 min. The lysates were centrifuged at 20 000 g for 10 min at 4°C. The supernatants were incubated with 10 μl of beads (cross-linked to 25 μg of monoclonal antibody, anti-FLAG, or anti-MTPAP complex) overnight at 4°C with rotation. Beads were washed 4 times, then boiled for 5 minutes after SDS loading was added. Finally, the IP fractions were analyzed by western blot analysis.

### Poly(A) tail length assay

The measurement of poly(A) tail length was performed as detailed elsewhere (68). Briefly, 3 μg of isolated total RNA from mutant and WT zebrafish were ligated to a phosphorylated oligonucleotide linker by T4 RNA ligase (Thermo Scientific) at 37°C for 3 h, according to the manufacturer's instructions. The ligated RNA was precipitated and cDNA synthesis was performed using the PrimeScript™ RT Reagent Kit (Takara) with a primer complementary to the linker sequence (anti-linker). A First round of PCR (29 cycles) was carried out using the anti-linker and a gene-specific upper primer. Then PCR products were purified using a PCR purification kit (Qiagen), followed by a second round of 12-cycle PCR using an inner anti-linker primer and a gene-specific lower primer. Finally, the resultant PCR products were assessed by 3% agarose gel electrophoresis or cloned into pGEM-T Easy Vector (Promega) for sequencing. The oligodeoxynucleotides for this analysis were listed in Supplemental Table S1.

### Statistical analysis

Statistical analysis was performed by unpaired, two-tailed Student's *t*-test, using GraphPad Prism 5 (version 5.0). Differences were considered significantly at a *P* < 0.05 (*P < 0.05, **P < 0.01 and ***P < 0.001).

## RESULTS

### Conservation of sequence and expression patterns of zebrafish Mto1

Zebrafish Mto1 comprises 659 amino acids containing the typical mitochondrial target signal sequence with 17 amino acids. The protein alignments of Zebrafish Mto1 with its homologs of other organisms, including *Homo sapiens, Mus musculus, Bos Taurus, Canis lupus, Rattus norvegicus* and *Caenorhabditis elegans* revealed an extensive conservation of protein sequence (Supplementary Figure S1). In particular, zebrafish Mto1 is 52.4% and 52.7% identical to the amino acid sequence of mouse and human MTO1, respectively.

**Figure 1.**
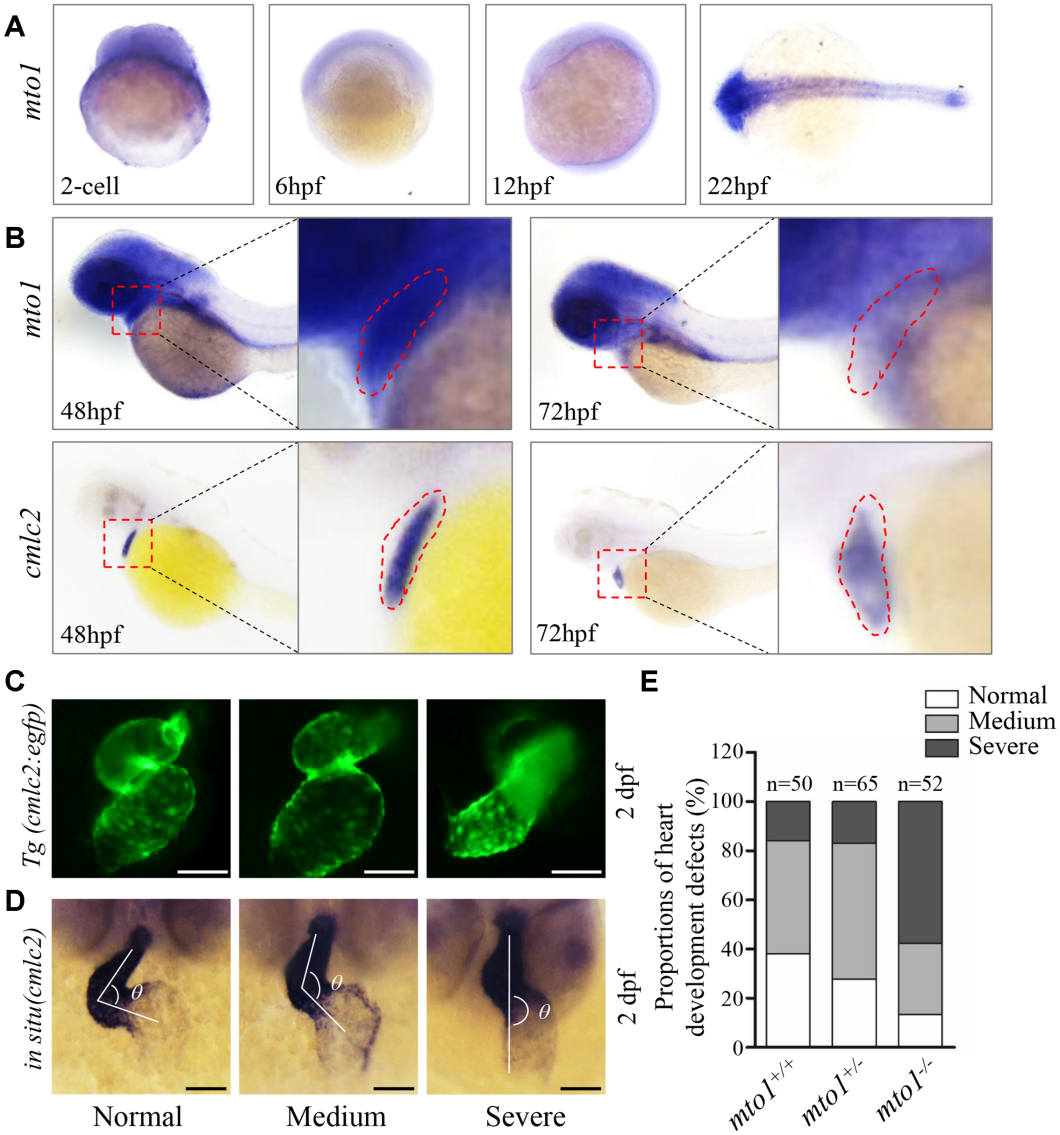
Heart development defects in zebrafish. (**A**) Whole-mount in situ hybridization (WISH) analysis of *mto1* expression on wild type larval zebrafish at various ages (2-cell to 24 hpf). (**B**) WISH analysis of *mto1* on wild type larval zebrafish at 48 hpf and 72 hpf. *cmlc2* marker was used to indicate the place of heart. Insets show higher magnifications of heart. (**C**) *Tg (cmlc2:egfp)* embryos at 2 dpf shows the heart-restricted GFP expression in both chambers. Scale bars: 50 μm. (**D**) WISH against *cmlc2*. According to the looping state, measured by the angles between ventricle and atrium, hearts were divided into normal (α < 90°), medium (90° < α < 180°), and severe (α > 180°). Scale bars: 50 μm. (**E**) The proportions of each phenotypes in the embryos in mutant and WT zebrafish.

The spatial patterns of *mto1* expression in zebrafish larvae were analyzed by whole amount *in situ* hybridization using DIG-labeled antisense probes. As shown in Figure 1A, the hybridization signals of *mto1* were detectable at two cell stages, indicating that *mto1* is maternally expressed. As shown in Figure 1B, the larval heart at 2 dpf displayed much higher expression levels of *mto1* than those in the larval heart at 3 dpf, while the expression levels of *mto1* in the larval brain at 2 dpf were comparable with those in 3 dpf larvae. However, the *cmlc2* (heart marker) was highly expressed in the heart at 2 dpf and 3 dpf. These data implied that *mto1* may play the important roles in the heart development.

### Generation of *mto1* knock-out zebrafish using CRISPR/Cas9 system

To explore the pathological consequences of *mto1* mutation, we used the CRISPR/Cas9 technology to generate zebrafish mutant lines where the Mto1 ortholog was disrupted. As shown in Supplementary Figure S2A, a single guide RNA (sgRNA) targeting exon 3 of *mto1* was injected into wild type one-cell stage embryos together with Cas9 mRNA. As a result, an allele, *mto1^del7bp^* was generated by introducing a 7 bp deletion in the exon 3 of *mto1* (heterozygous and homozygous zebrafish were described as *mto1*^+/−^, *mto1*^−/−^ and wild type as WT). In fact, this deletion caused a frameshift from codon 115 and the introduction of a premature stop at codon 135 (Supplementary Figure S2A, B). This allele was subsequently propagated after confirmation of the mutation by Sanger sequencing, DNA-PAGE and Western blot analyses (Supplementary Figure S2B-D). Both *mto1*^+/−^ and *mto1*^−/−^ zebrafishes were adult-viable.

### Loss of Mto1 altered embryonic heart development

Heart was the first organ to form and function during zebrafish embryogenesis (69,70). Cardiac primordium went through migration and left-jogging process within the first day post fertilization and initiated contraction around 1 dpf. The chambers of ventricle and atrium formed and the whole heart underwent an S-looping process at 2 dpf. Finally, the functional heart was formed at 5 dpf (71,72). Cardiac-specific marker *cmlc2* was used to investigate the effect of *mto1* deletion on heart development by either green fluorescence or whole-mount *in situ* hybridization at 2 dpf. As shown in Figure 1C, *mto1*^−/−^ zebrafish exhibited various degrees of perturbed S-looping processing. Based on looping angles between ventricle and atrium, zebrafish hearts were divided into categories of normal (α < 90°), medium (90° < α < 180°), and severe (α > 180°) (Figure 1D). As shown in Figure 1E, 46% *mto1*^+/+^ (*n* = 50), 55% *mto1*^+/−^ (*n* = 65) and 29% *mto1*^−/−^ (*n* = 52) displayed mild-loop heart, respectively. In contrast, 16% *mto1*^+/+^ (*n* = 50), 17% *mto1*^+/−^ (*n* = 65) and 58% *mto1*^−/−^ (*n* = 52) had no-loop heart, respectively. These data indicated that the deletion of *mto1* resulted in defects in embryonic heart development.

### The *mto1* mutants exhibited hypertrophic cardiomyopathy in adult zebrafish.

**Figure 2.**
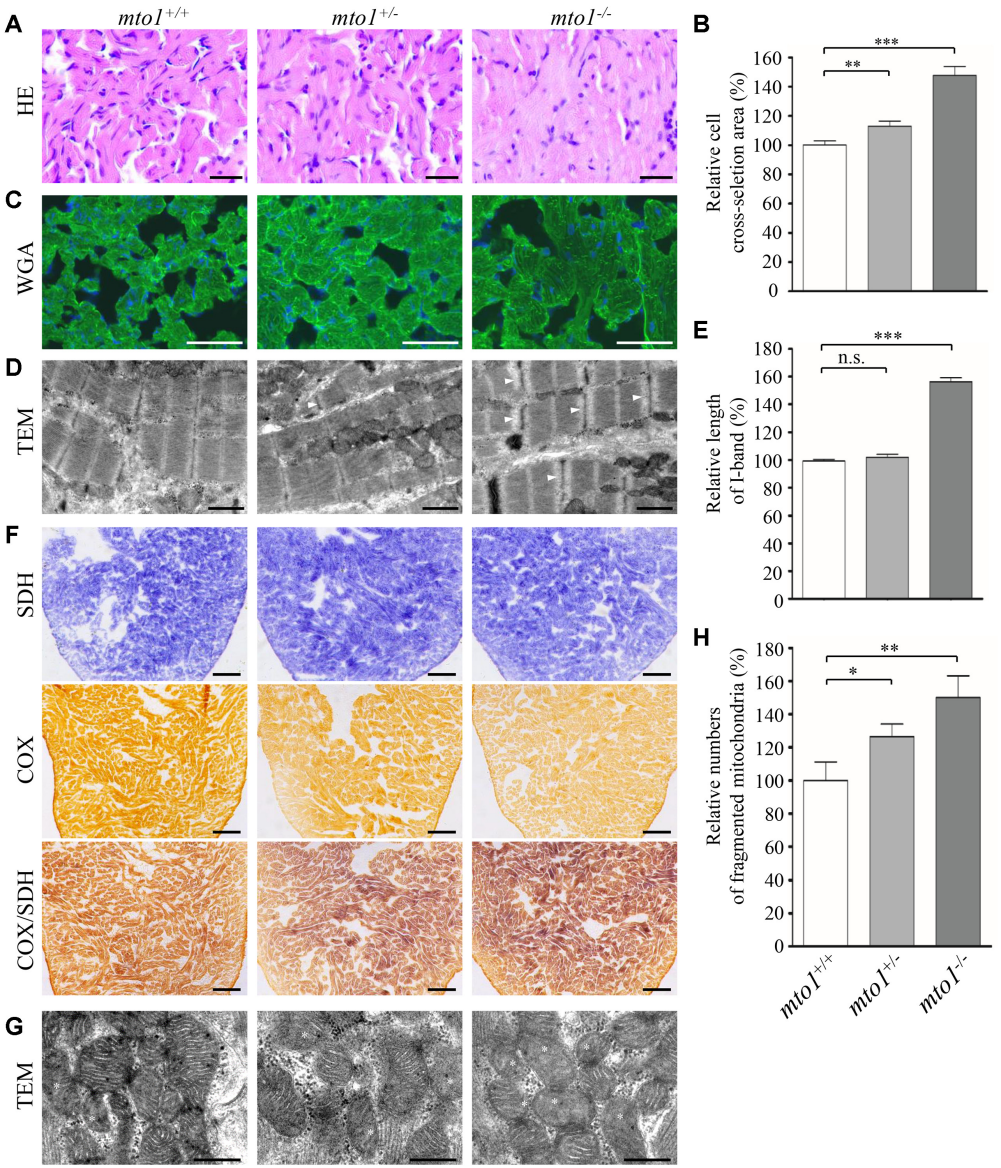
Hypertrophic cardiomyopathy and mitochondrial defects in the zebrafish. (**A**) Hematoxylin and eosin stained (H&E) histological sections of hearts from *mto1*^−/−^, *mto1*^+/−^ and *mto1^+/+^* zebrafish at the age of 6 months. Scale bars: 20 μm. (**B**) The relative cross-sectional areas of cardiomyocytes in the *mto1*^+/+^ (*n* = 104), *mto1*^+/−^ (*n* = 112), *mto1*^−/−^ (*n* = 92) zebrafishes, staining with H&E. (**C**) The cardiomyocytes from mutant and WT zebrafishes were visualized via WGA staining. Scale bars: 20 μm. (**D**) Ventricular heart muscle sections of transmission electron microscopy in the mutant and WT zebrafish. Ultrathin sections were visualized with 15 000× magnifications. Widened I-band in the sarcomere units were indicated by white arrowhead. Scale bars: 1 μm. (**E**) Quantification of the I-band lengthes of *mto1*^+/+^ (*n* = 28), *mto1*^+/−^ (*n* = 18) and *mto1*^−/−^ (*n* = 29) zebrafish. The values for the mutants were expressed as percentages of the mean values for the WT. (**F**) Assessment of mitochondrial functions in cardiomyocytes by enzyme histochemistry (EHC) staining for cytochrome c oxidase (COX) and succinate dehydrogenase (SDH) in the frozen-sections of ventricles in the *mto1*^−/−^, *mto1*^+/−^ and *mto1*^+/+^ zebrafish at six-month old. Scale bars: 50 μm. (**G**) Mitochondrial networks from cardiomyocytes of transmission electron microscopy. Ultrathin sections were visualized with 30 000× magnifications. Fragmented mitochondria are indicated by asterisks. Scale bars: 0.5 μm. (**H**) Quantifications of fragmented mitochondria numbers of cardiomyocytes from the *mto1*^−/−^, *mto1*^+/−^ and *mto1*^+/+^ zebrafish. The error bars indicate standard deviations of the means. *P* indicates the significance, according to Student's *t* test, of the difference between *mto1*^+/−^ or *mto1*^−/−^ and WT values, denoted by asterisks (*P< 0.05, **P< 0.01, ***P< 0.001), and non-significant differences by n.s.

We then investigated if *mto1*^−/−^ zebrafish recapitulated the hypertrophic cardiomyopathy phenotypes in the patients carrying the *MTO1* mutations. As shown in Figure 2A, cardiomyocytes of 6-month-old *mto1*^−/−^ zebrafish stained with H&E showed hypertrophy of cardiac myocytes and myocardial fiber disarray. The abnormal myocytes included the enlarged size, bizarre-shaped nuclei and disorganized patterns. As shown in Figure 2B, the cross-sectional areas of cardiomyocytes in *mto1*^−/−^ and *mto1*^+^^/−^ zebrafishes were 148% (*P* < 0.0001) and 113% (*P* = 0.0048), relative to the mean values measured in WT zebrafish. As illustrated in Figure 2C, these phenotypes were verified by direct staining the outline of cardiomyocyte by FITC-conjugated WGA (56). As shown in Figure 2D, the *mto1*^+/−^ and *mto1*^−/−^ zebrafishes exhibited defects in cardiac myofibrils and widened I-bands using transmission electron micrographs. As shown in Figure 2E, the lengths of I-bands were significantly increased in the *mto1^−^^/^^−^ and mto1^+/−^* zebrafishes, as compared with those in WT zebrafish. These data demonstrated that *mto1^−^^/−^* zebrafish recapitulated the clinical phenotypes in the HCM patients carrying the *MTO1* mutation(s).

### Loss of *mto1* caused mitochondrial defects in cardiomyocytes

Mitochondrial dysfunctions in cardiomyocytes were assessed by enzyme histochemical assays for SDH, COX and COX/SDH in the frozen-sections of ventricles of *mto1^−^^/^^−^, mto1^+/−^* and WT zebrafishes at 6 months old. As shown in Figure 2F, marked reductions of COX activity but no change of SDH activity were observed in *mto1^−^^/^^−^* zebrafish, as compared to the WT zebrafish. A sequential COX-SDH histochemical assay confirmed a generalized decrease in COX reactivity in the *mto1^−^^/^^−^* zebrafish hearts. Mitochondrial defects in cardiomyocytes of mutant and WT zebrafish at 6 months old were further evaluated by using transmission electron microscope. As shown in Figure 2G, cardiomyocytes of *mto1^−^^/^^−^* mutant zebrafish displayed abnormal mitochondrial morphology including fragmented mitochondria and partial loss of cristae, as compared to those of WT zebrafish. As shown in Figure 2H, fragmented mitochondria per cardiomyocyte cell in the *mto1^−^^/^^−^* (*n* = 32) and *mto1^+/^^−^* (*n* = 30) zebrafish were 126% and 150%, related to the average values of those in the WT zebrafish (*n* = 28).

### Analysis of nucleotide modifications and steady-state levels of mitochondrial tRNAs

**Figure 3.**
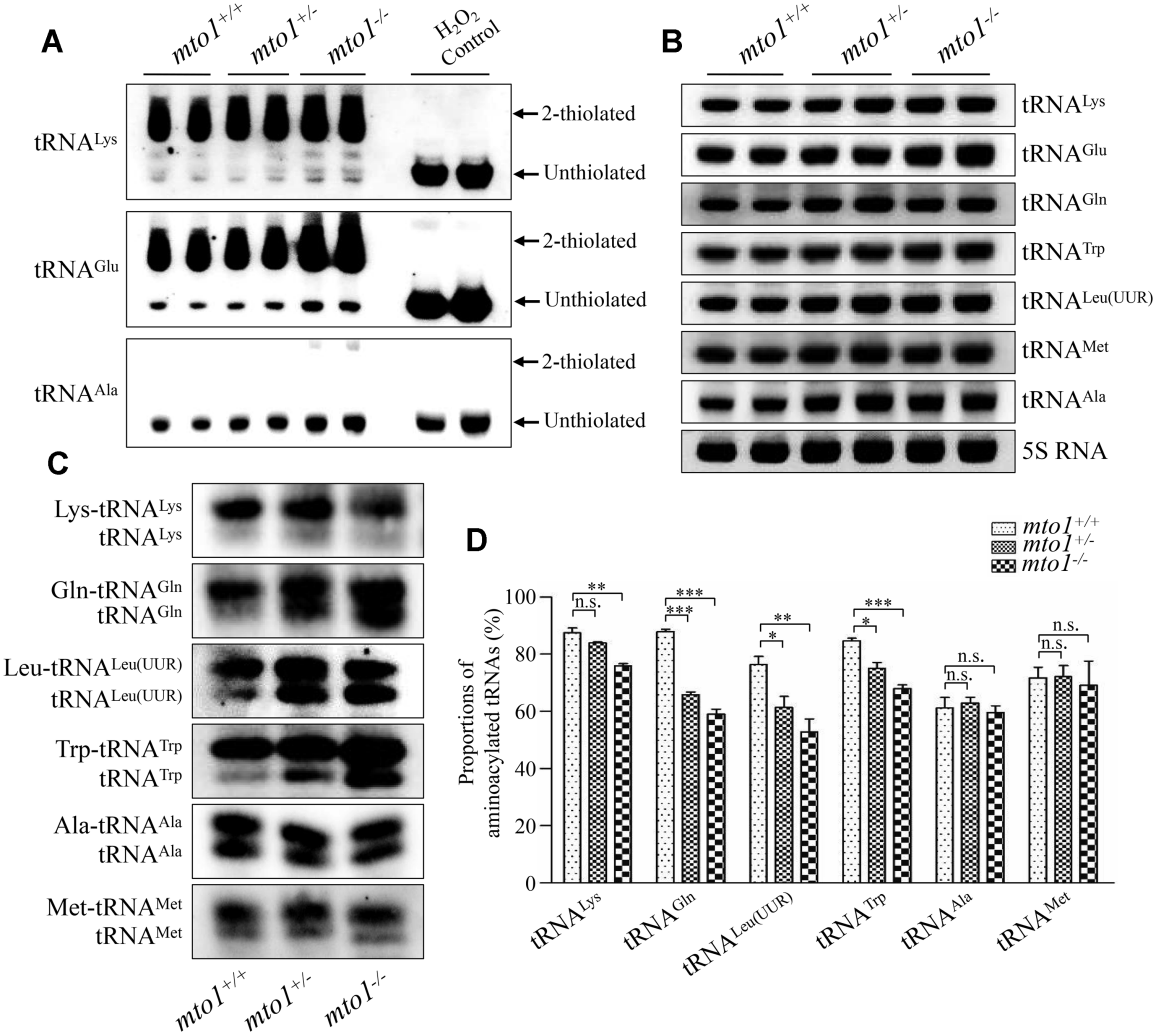
Northern blotting analysis of mitochondrial tRNA. (**A**) APM gel electrophoresis. Five μg total RNAs of mutant and WT zebrafish were separated by polyacrylamide gel electrophoresis that contains 0.05 mg/ml APM, electroblotted onto a positively charged membrane, and hybridized with DIG-labeled oligonucleotide probes specific for tRNA^Lys^, tRNA^Glu^ and tRNA^Ala^, respectively. H_2_O_2_-treated tRNA was used as a control of complete oxidation of thiolation, eliminating the mobility shift. The retarded bands of 2-thiolated tRNAs and non-retarded bands of tRNA without thiolation are marked by arrows. (**B**) Northern blot analysis of tRNA under a denaturing condition. Five micrograms of total RNAs of mutant and WT zebrafish were electrophoresed through a denaturing polyacrylamide gel, electroblotted and hybridized with the DIG-labeled oligonucleotide probes specific for tRNA^Lys^, tRNA^Glu^, tRNA^Gln^, tRNA^Trp^, tRNA^Leu(UUR)^, tRNA^Met^, tRNA^Ala^, and 5S rRNA as a loading control, respectively. (**C**) Five micrograms of total RNAs from mutant and WT zebrafish under acid conditions were electrophoresed at 4°C through an acid (pH 5.2) 10% polyacrylamide with 8 M urea gel, electroblotted, and hybridized with a DIG-labeled oligonucleotide probes specific for the tRNA^Lys^, tRNA^Gln^, tRNA^Leu(UUR)^, tRNA^Trp^, tRNA^Ala^ and tRNA^Met^, respectively. (**D**) Quantification of aminoacylated proportions of tRNAs in mutant and WT zebrafish. The calculations were based on three independent experiments. Graph details and symbols are explained in the legend to the Figure 2.

To further examine whether the loss of Mto1 affected the nucleotide modifications at position 34 of tRNAs in zebrafish, the 2-thiouridylation levels of tRNAs were determined by isolating total RNAs from WT, *mto1*^+/−^ and *mto1*^−/−^ zebrafishes, quantifying the 2-thiouridine modification by the retardation of electrophoresis mobility in APM polyacrylamide gel, and hybridizing DIG-labeled probes for mitochondrial tRNA^Lys^, tRNA^Glu^ and tRNA^Ala^, using H_2_O_2_-treated tRNAs as internal controls (30,53,56). In this system, the mercuric compound specifically interacted with the tRNA^Lys^ and tRNA^Glu^ containing the thiocarbonyl group, but not tRNA^Ala^ lacking this modification, thereby retarding tRNA migration. As shown in Figure 3A and supplemental Figure S3A, 2-thiouridylation levels of mitochondrial tRNA^Lys^ and tRNA^Glu^ in the *mto1*^+/−^ and *mto1*^−/−^ zebrafish were comparable with those in the WT zebrafish.

To assess if the *mto1* mutation altered the stability of tRNAs, we subjected total RNAs from mutant and WT zebrafishes to Northern blotting and hybridized them with DIG-labeled oligodeoxynucleotide probes tRNA^Lys^, tRNA^Glu^, tRNA^Gln^, tRNA^Trp^, tRNA^Leu(UUR)^, tRNA^Met^ and tRNA^Ala^ with 5S rRNA as a loading control, respectively (Figure 3B). For comparison, the levels of each tRNA were normalized to the reference 5S rRNA. The steady-state levels of tRNA^Lys^, tRNA^Glu^, tRNA^Gln^, tRNA^Trp^, tRNA^Leu(UUR)^, tRNA^Met^ and RNA^Ala^ in *mto1*^+/−^ and *mto1*^−/−^ zebrafish were comparable with those in WT zebrafish (Supplementary Figure S3B).

### Deficient aminoacylation of mitochondrial tRNAs

To evaluate whether the loss of Mto1 affected the aminoacylation of tRNAs, we examined the aminoacylation levels of tRNA^Lys^, tRNA^Gln^, tRNA^Leu(UUR)^, tRNA^Trp^ with taurine modification and tRNA^Ala^ and tRNA^Met^ without taurine modification by using electrophoresis in an acidic urea PAGE system to separate uncharged tRNA species from the corresponding charged tRNA, electroblotting and hybridizing with the tRNA probes described above. As shown in Figure 3C, *mto1*^−/−^ and *mto1*^+/−^ zebrafish exhibited significant decreases in the aminoacylated levels of mitochondrial tRNAs, as compared with those in WT zebrafish. As shown in Figure 3D, the levels of aminoacylated tRNA^Lys^, tRNA^Gln^, tRNA^Leu(UUR)^ and tRNA^Trp^ in the *mto1*^−/−^ zebrafish were 87%, 67%, 69% and 80% of those in WT zebrafish, while the efficiencies of aminoacylated tRNA^Lys^, tRNA^Gln^, tRNA^Leu(UUR)^ and tRNA^Trp^ in the *mto1*^+/−^ zebrafish were 96%, 75%, 80% and 88% of those in WT zebrafish. However, the efficiencies of aminoacylated tRNA^Ala^ and tRNA^Met^ in the *mto1*^+/−^ and *mto1*^−/−^ zebrafish were comparable with those in WT zebrafish.

### Altered conformation of mitochondrial tRNAs

**Figure 4.**
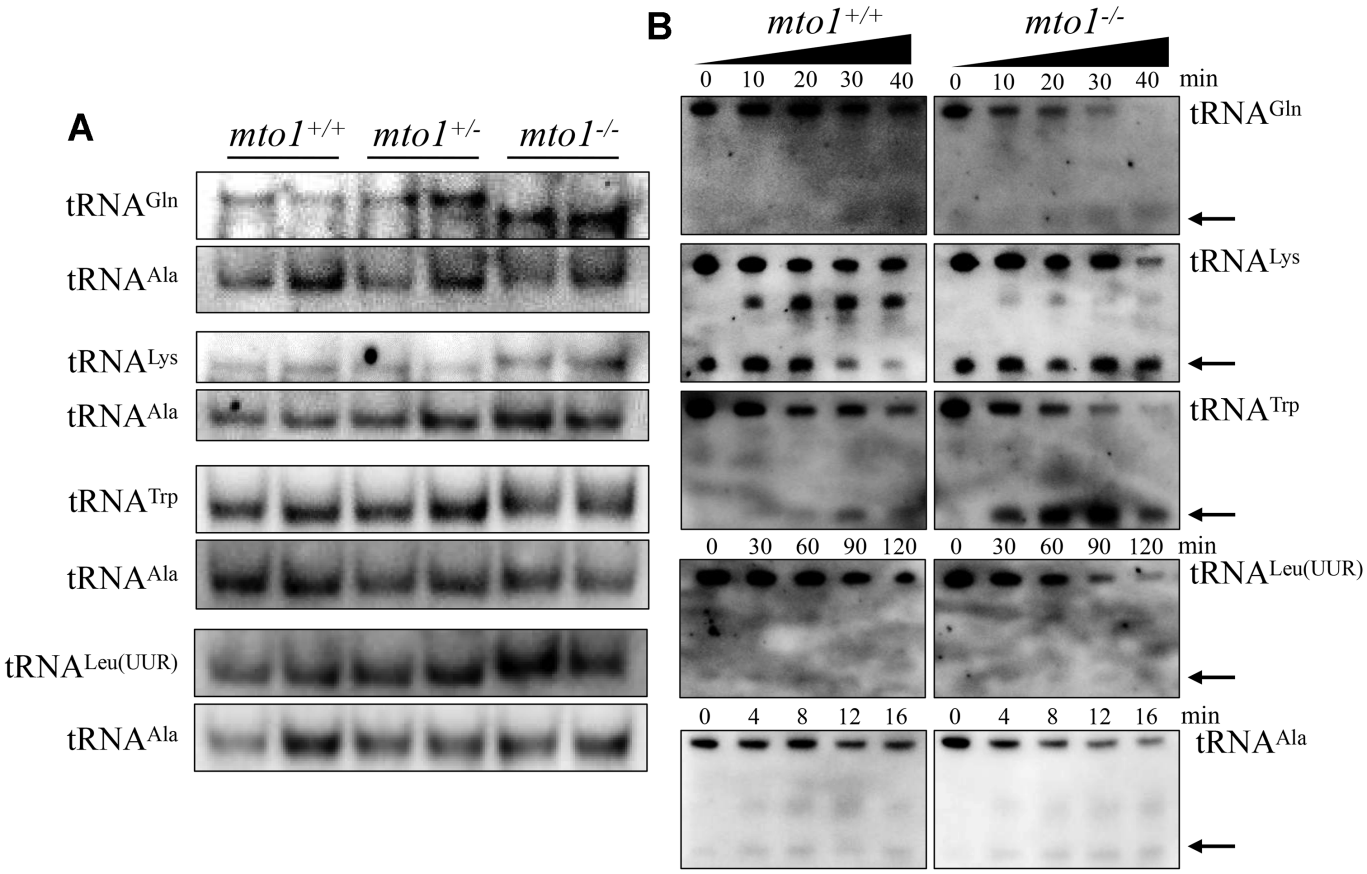
Analysis of mitochondrial tRNA conformation. (**A**) Northern blot analysis of tRNAs under native condition. Five micrograms of total RNAs from mutant and WT zebrafish were electrophoresed through a native polyacrylamide gel, electroblotted and hybridized with the DIG-labeled oligonucleotide probes as tRNA^Gln^, tRNA^Trp^, tRNA^Leu(UUR)^, tRNA^Lys^ and tRNA^Ala^, respectively. (**B**) S1 digestion patterns of tRNA^Gln^, tRNA^Trp^, tRNA^Leu(UUR)^, tRNA^Lys^ and tRNA^Ala^. Two micrograms of RNAs from *mto1*^−/−^ and WT zebrafish were used for the S1 cleavage reaction at various lengths (from 0 to 120 min). Cleavage products of tRNAs were resolved in 10% denaturating PAGE gels with 8 M urea, electroblotted and hybridized with 3′ end DIG-labeled oligonucleotide probes specific for tRNA^Gln^, tRNA^Trp^, tRNA^Leu(UUR)^, tRNA^Lys^ and tRNA^Ala^, respectively. Arrows denoted the 30–40 nt regions showing 3′ tRNA half.

Nucleotide modifications play a critical role in forming functional structure of tRNAs (73). To investigate if that the deficient nucleotide modification affected the conformation of mitochondrial tRNA, total RNAs from WT and mutant zebrafishes were electrophoresed through 10% polyacrylamide gel (native condition) in Tris-glycine buffer and then electroblotted onto a positively charged nylon membrane for hybridization analysis with DIG-labeled oligodeoxynucleotide probes for tRNA^Gln^, tRNA^Lys^, tRNA^Trp^, tRNA^Leu(UUR)^ and tRNA^Ala^, respectively. As shown in Figure 4A, the electrophoretic patterns under native condition showed that the tRNA^Gln^ in *mto1*^−/−^ zebrafish migrated faster than those of WT zebrafish, while tRNA^Lys^, tRNA^Trp^ and tRNA^Leu(UUR)^ in *mto1*^−/−^ zebrafish migrated slightly slower than those in WT zebrafish. However, there were no obvious electrophoretic mobility differences of the tRNA^Ala^ without taurine modification between *mto1*^−/−^ and WT zebrafish.

We further evaluated whether the loss of Mto1 perturbed the structures of tRNAs by analyzing the sensitivity of tRNA^Gln^, tRNA^Lys^, tRNA^Trp^ and tRNA^Leu(UUR)^ (present taurine modification in WT) as well as tRNA^Ala^ (absent taurine in WT), from *mto1*^−/−^ and WT zebrafish to digestion with the nuclease S1. The resultantly digested-products from *mto1*^−/−^ and WT zebrafish were then followed by Northern blot analysis using tRNA probes that hybridized only to 3′ half tRNAs. As illustrated in Figure 4B, the tRNA^Gln^, tRNA^Lys^, tRNA^Trp^ and tRNA^Leu(UUR)^ from *mto1*^−/−^ zebrafish were more sensitive to S1-mediated digestion than those from WT zebrafish and exhibited remarkable differences in S1-mediated digestion patterns of tRNAs from *mto1*^−/−^ zebrafish. As shown in Supplemental Figure S4, the ratios of tRNA half/full length tRNA^Lys^, tRNA^Trp^, tRNA^Gln^ and tRNA^Leu(UUR)^ increased significantly between *mto1*^−/−^ and WT zebrafish, respectively. Conversely, there was no significant difference between the sensitivity of tRNA^Ala^ from *mto1*^−/−^ and WT zebrafish to digestion with the nuclease S1. These data validated that the inactivation of Mto1 changed the conformation of mitochondrial tRNAs.

### Reductions in the levels of mitochondrial proteins

**Figure 5.**
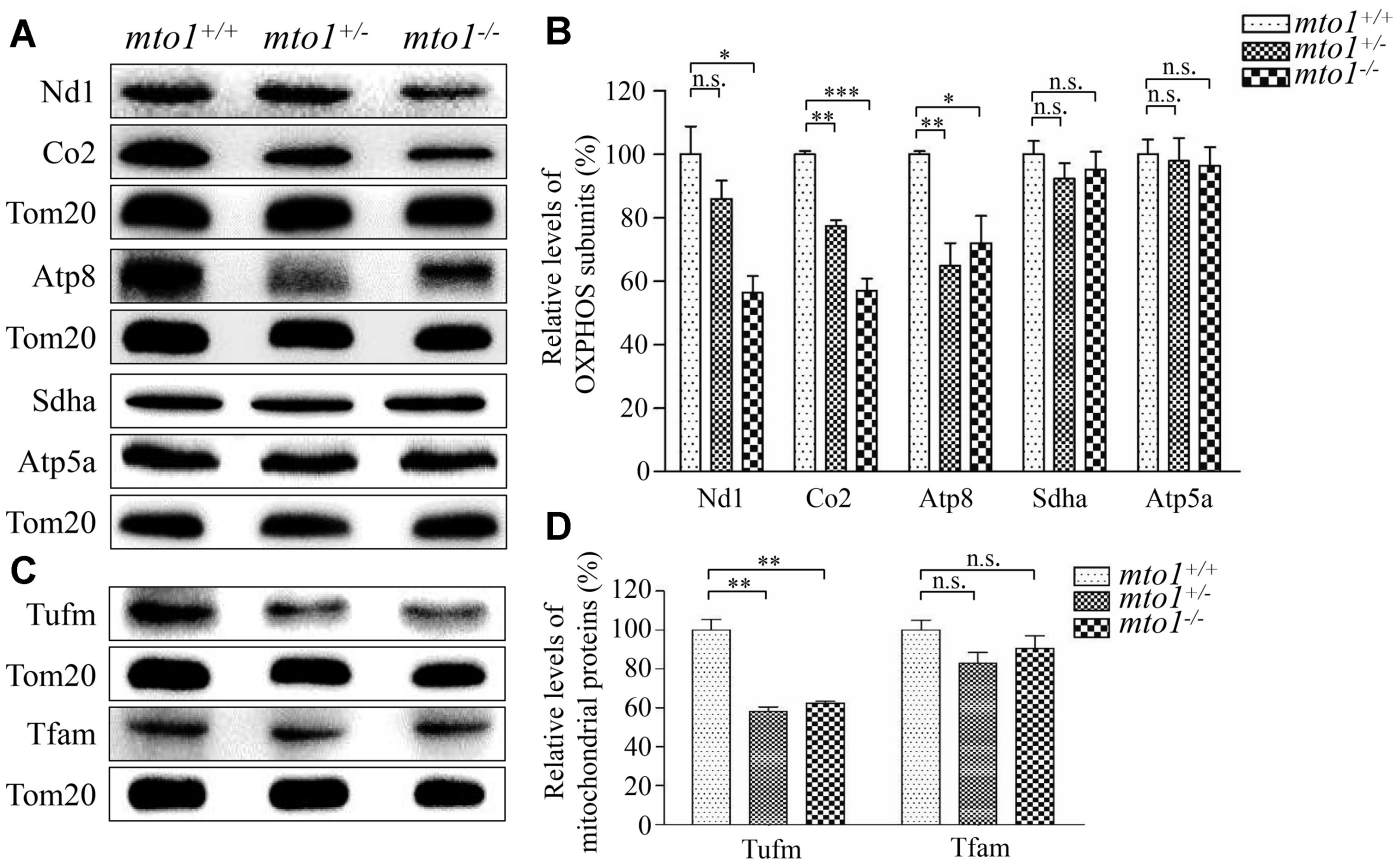
Western blotting analysis of mitochondrial proteins. (**A**, **C**) Twenty micrograms of total proteins from mutant and WT zebrafish were electrophoresed through a denaturing polyacrylamide gel, electroblotted and hybridized with antibodies for 5 subunits of OXPHOS (mitochondrion-encoding Nd1, Co2 and Atp8 and nucleus-encoding Sdha and Atp5a), Tufm, Tfam and Tom20 as a loading control. Quantification of levels of OXPHOS subunits (**B**) and other mitochondrial proteins (**D**). Average contents of Nd1, Co2, Atp8, Atp5a, Sdha, Tufm and Tfam were normalized to the average content of Tom20 in mutant and WT zebrafish. The values for the mutant zebrafish are expressed as percentages of the average values for the WT zebrafish. The calculations were based on three independent determinations. Graph details and symbols are explained in the legend to Figure 2.

To examine whether the aberrant mitochondrial tRNA metabolism impaired mitochondrial translation, western blot analysis was carried out to examine the five subunits of OXPHOS [mitochondrion-encoding Nd1 (subunit 1 of NADH dehydrogenase), Co2 (subunit 2 of cytochrome c oxidase) and Atp8 (subunit of H^+^-ATPase), and nucleus-encoding Atp5a (subunit of H^+^-ATPase) and Sdha (subunit of succinate dehydrogenase)], and 2 nucleus-encoding mitochondrial proteins [Tufm (mitochondrial Tu translation elongation factor) and Tfam (mitochondrial transcription factor A)] in mutant and WT zebrafish using Tom20 as a loading control. As shown in Figure 5A, C, the levels of Nd1, Co2, Atp8 and Tufm were significantly decreased in mutant zebrafish, as compared with those in WT zebrafish. However, the levels of Sdha, Atp5a and Tfam did not differ significantly between *mto1*^−/−^ and WT zebrafish, respectively. As shown in Figure 5B,D, the levels of Nd1, Co2, Atp8 and Tufm in *mto1*^−/−^ zebrafish were 56%, 57%, 72% and 62%, relative to the mean values measured in the WT zebrafish (*P* = 0.0004–0.0309), respectively. Furthermore, the levels of Nd1, Co2, Atp8 and Tufm in *mto1*^+/−^ zebrafish were 86%, 77%, 65% and 58%, relative to the average values in WT zebrafish (*P* = 0.0005–0.2501), respectively. These data implied that the loss of Mto1 primarily affected mitochondrial translation rather than transcription.

### Altered stability and activities of OXPHOS complexes

**Figure 6.**
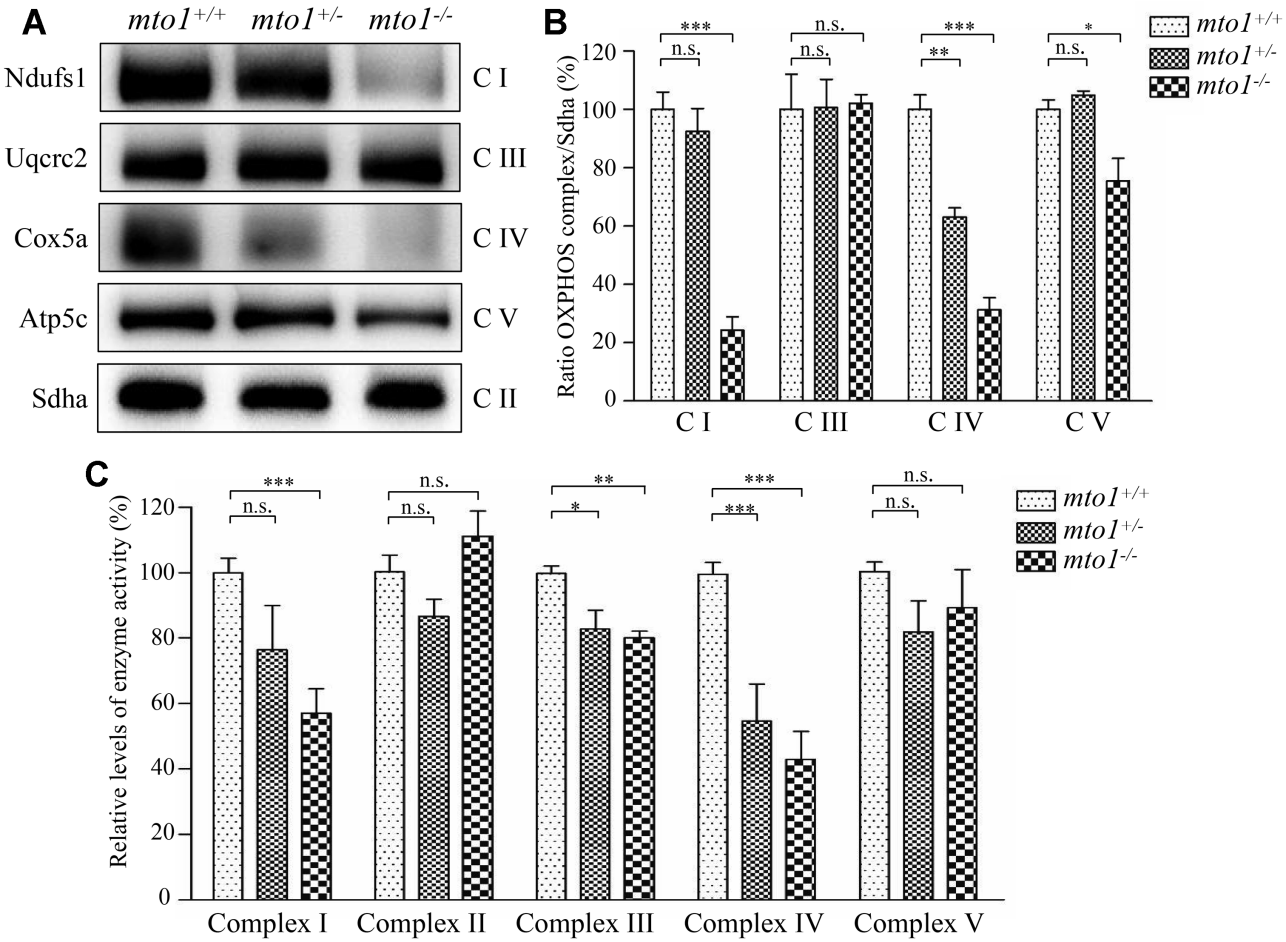
The instability of OXPHOS complexes. (**A**) The steady-state levels of five OXPHOS complexes by Blue-Native gel electrophoresis. Fifteen micrograms of mitochondrial proteins from mutant and WT zebrafish were electrophoresed through a Blue-Native gel, electroblotted and hybridized with antibodies for Ndufs1, Uqcrc2, Cox5a, Atp5c (subunits of complex I, III, IV and V, respectively) as well as Sdha (subunit of complex II) as a loading control. (**B**) Quantification of levels of complexes I, III, IV and V in mutant and WT zebrafish. (**C**) The activities of OXPHOS complexes were investigated by enzymatic assays on complexes I, II, III, IV and V in mitochondria isolated from mutant and WT zebrafish. The calculations were based on three independent determinations. Graph details and symbols are explained in the legend to Figure 2.

To assess if the *mto1* mutation-induced alterations affected the stability of OXPHOS complexes, we measured the steady-state levels of five OXPHOS complexes by Blue-Native gel electrophoresis using OXPHOS-subunit specific antibodies – Ndufs1 (complex I), Uqcrc2 (complex III), Cox5a (complex IV), Atp5c (complex V) and Sdha (complex II) as a loading control. As shown in Figure 6A,B, the levels of complex I, complex IV and complex V in the *mto1*^−/−^ zebrafish were 24%, 31% and 75%, relative to the mean values measured in the WT zebrafish. Furthermore, the levels of complex I, complex IV and complex V in *mto1*^+/−^ zebrafish were 92%, 63% and 104%, relative to the mean values measured in the WT zebrafish. However, the levels of complex III in *mto1*^−/−^ and *mto1*^+/−^ were comparable with those in WT zebrafish.

To further investigate the effect of *mto1* mutation on the oxidative phosphorylation, we measured the activities of OXPHOS complexes by isolating mitochondria from WT, *mto1*^+/−^ and *mto1*^−/−^ zebrafish. Complex I activity was determined by following the oxidation of NADH with ubiquinone as the electron acceptor (64,65). The activity of complex II (succinate ubiquinone oxidoreductase), which was exclusively encoded by the nuclear DNA, was examined by the artificial electron acceptor DCPIP. Complex III (ubiquinone cytochrome c oxidoreductase) activity was measured as the reduction of cytochrome c (III) using d-ubiquinol-2 as the electron donor. Complex IV (cytochrome c oxidase) activity was monitored by following the oxidation of cytochrome c (II). Complex V (F_1_, F_0_-ATP synthetase) activity was measured as the oxidation of NADH using phosphoenolpyruvate as the electron acceptor. As shown in Figure 6C, the activities of complex I, III and IV in *mto1*^−/−^ zebrafish were 57%, 80% and 43%, relative to the mean values measured in the WT zebrafish, respectively. Furthermore, the activities of complex I, III and IV in *mto1*^+/-^ zebrafish were 76%, 83% and 55% of the average values measured in WT zebrafish, respectively. However, the activities of complex II and complex V in *mto1*^−/−^ and *mto1*^+/−^ zebrafish were comparable with those in WT zebrafish. These data indicated that the loss of *mto1* caused the pronounced defects of complexes I and IV.

### Transcriptome analysis of *mto1*^KO^ and WT zebrafish hearts

**Figure 7.**
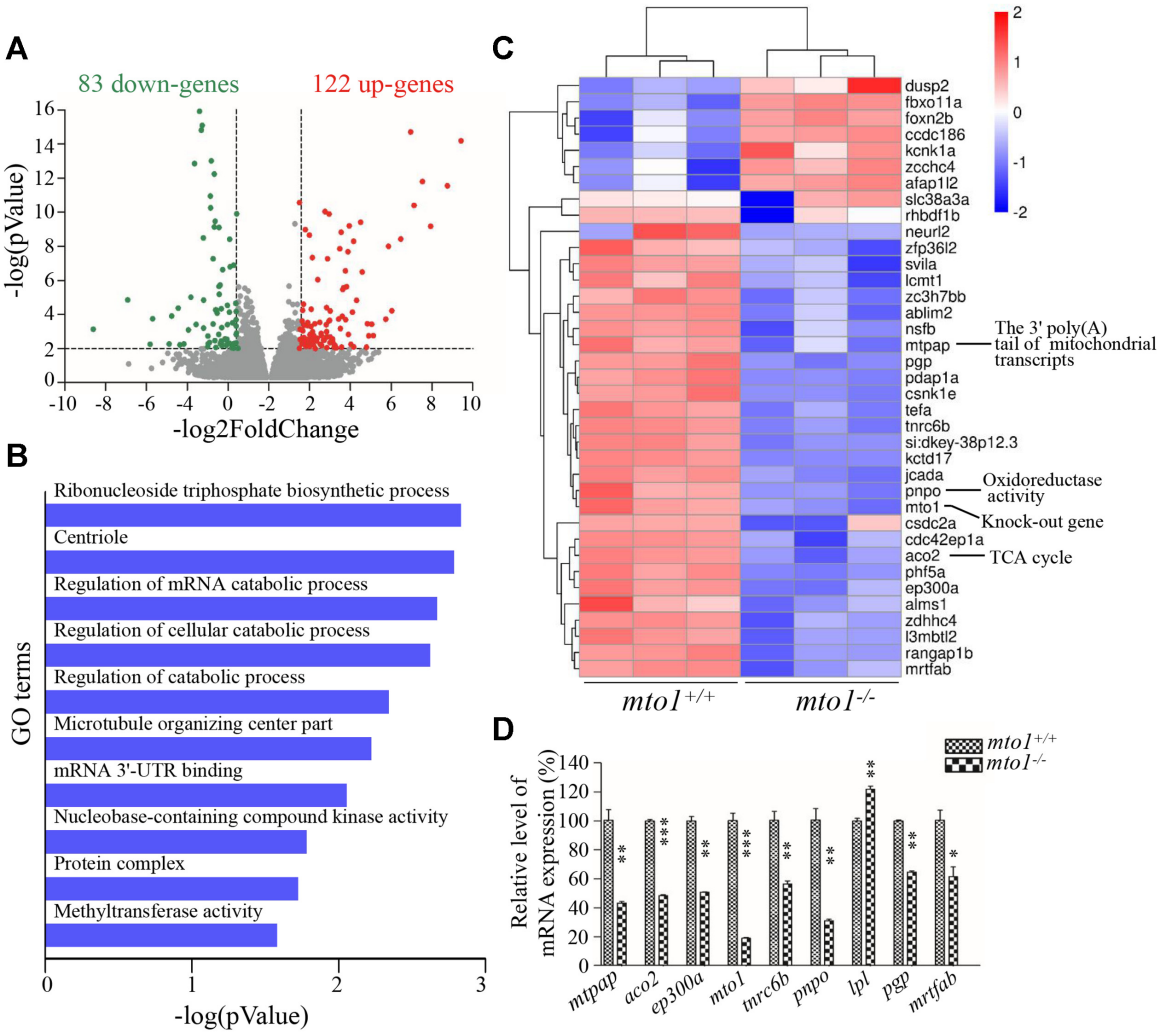
Transcriptome analysis of zebrafish hearts. (**A**) Volcano plots of total RNA sequencing revealed 205 differentially expressed genes (122 up-regulated (red dots) and 83 down-regulated (green dots)) between WT and *mto1*^−/−^ zebrafish hearts (*n* = 5) at the age of 4–6 months. (**B**) Gene ontology (GO) enrichment analysis of *mto1* target genes. The color of the bars indicates the *P*-value for the gene enrichment in our analysis. (**C**) Hierarchical clustering of the top 37 differentially expressed genes are presented as a heat map. Higher and lower expressed genes were marked in red and blue, respectively. (**D**) PCR validation of selected differentially regulated genes. Graph details and symbols are explained in the legend to Figure 2.

To examine if the MTO1-deficiency changed the gene expression profile for the development of hypertrophic cardiomyopathy and the deficiency of complex I and IV, we performed mRNA sequencing on hearts of WT and *mto1*^−/−^ zebrafish at the age of 4–6 months. As shown in Figure 7A, transcriptome analysis of *mto1*^−/−^ zebrafish hearts detected the deregulation of 205 genes (*P*-value < 0.01 and |log_2_ (fold change)| > 1.58), including 122 up-regulated genes and 83 down-regulated genes, as compared with those in the WT zebrafish hearts. As shown in Figure 7B, gene ontology analysis indicated that deregulated genes were enriched (top 10 GO classes) in the pathways involving in ribonucleoside triphosphate biosynthetic process, centriole, regulation of mRNA catabolic process, regulation of cellular catabolic process, regulation of catabolic process, microtubule organizing center part, mRNA 3'-UTR binding, nucleobase-containing compound kinase activity, protein complex, and methyltransferase activity in the *mto1*^−/−^ zebrafish hearts, as compared with those in WT zebrafish hearts. As shown in Figure 7C, the heat map showed the representation of top 37 differentially expressed genes between *mto1*^−/−^ and WT zebrafish, including genes involved in mitochondrial biogenesis, including *mtpap* [mitochondrial poly(A) polymerase], *pnpo* (pyridoxamine 5'-phosphate oxidase) and *aco2* (aconitase 2, mitochondria). These results were further confirmed by real time quantitative PCR (RT-qPCR) analyses of selected genes: *mtpap*, *aco2*, *ep300a*, *mto1*, *tnrc6b* (trinucleotide repeat containing 6b), *pnpo*, *lpl* (lipoprotein lipase), *pgp* (phosphoglycolate phosphatase) and *mrtfab* (myocardin related transcription factor Ab) (Figure 7D). In particular, it has been showed that *MTPAP* mutations caused the defects of complexes I and IV (14), similar to biochemical defects in the cells carrying *MTO1* mutations (36,39). Furthermore, *mtpap* revealed the similar expression patterns to those of *mto1* in the zebrafish embryos. As shown in Supplementary Figure S5, the *mtpap* in the larvae heart at 2 dpf displayed much higher expression levels than those in larvae heart at 3 dpf. These imply that Mtpap may interact with Mto1 to maintain the stability of oxidative phosphorylation complexes.

### *mto1* ^−/−^ zebrafish exhibited abnormal polyadenylation of mitochondrial mRNAs by interacting with Mtpap

**Figure 8.**
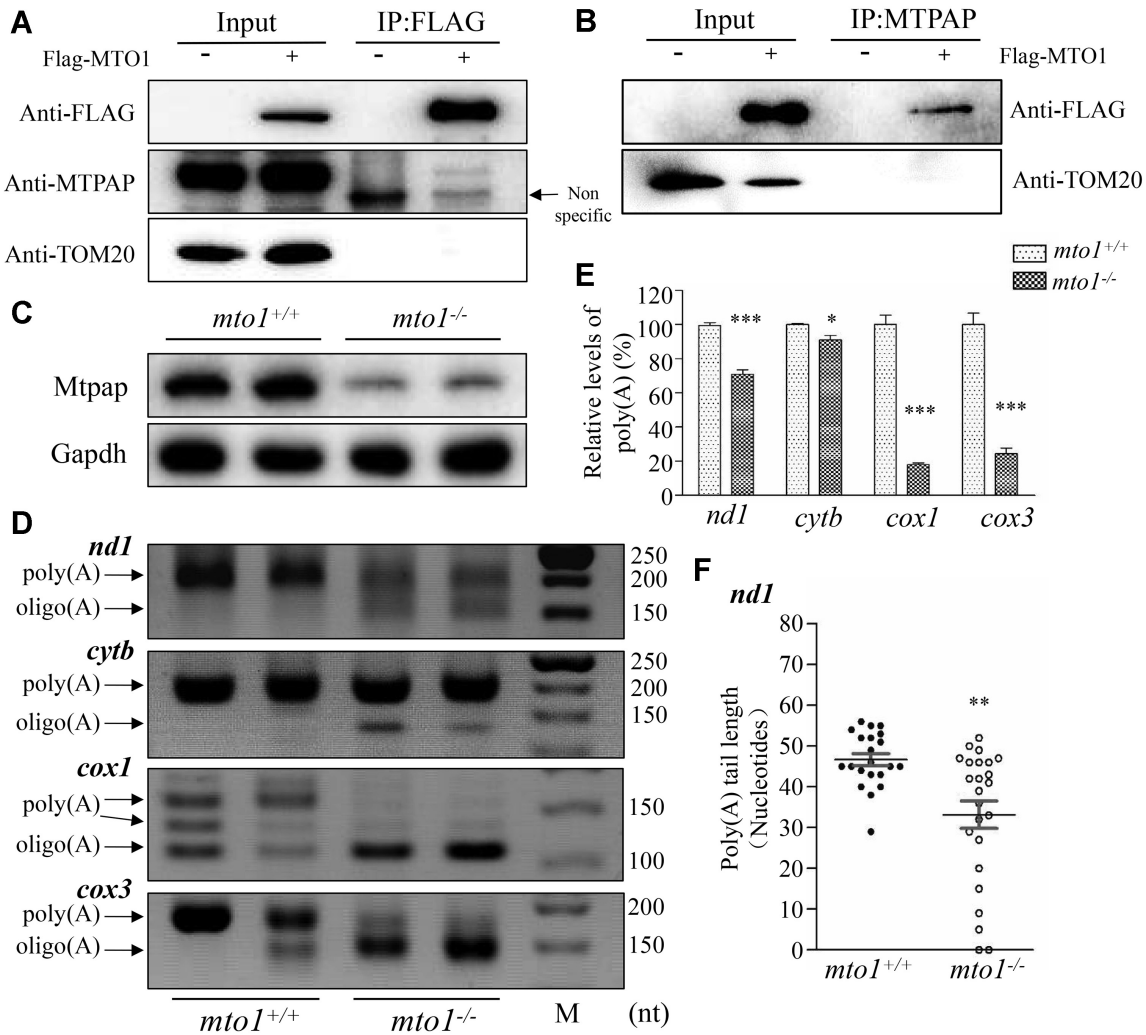
The synergic effects between Mto1 and Mtpap on the polyadenylation of mRNAs. (**A, B**) Immunoprecipitation analysis of MTO1 with MTPAP. HEK 293T cells transiently expressing with or without MTO1-FLAG were solubilized with a lysis buffer and lysate proteins were immuno-precipitated with immunocapture buffer (left) (input) and FLAG-antibody (right) (IP), respectively. Immunoprecipitates were analyzed by SDS-PAGE and Western blotting using anti-FLAG, anti-MTPAP and TOM20 antibodies, respectively. (**C**) Western blot analysis. Twenty micrograms of total proteins from *mto1*^−/−^ and WT zebrafish were electrophoresed through a denaturing polyacrylamide gel, electroblotted and hybridized with antibodies for Mtpap and Gapdh as a loading control. (**D**) Polyadenylation profiles of mitochondrial mRNAs upon *mto1*^−/−^ and WT zebrafish hearts. The 3′ termini of *nd1, cytb*, *cox1* and *cox3* mRNAs were assessed by RT-PCR amplifications from polyadenylated and oligoadenylated RNAs of *mto1*^−/−^ and WT zebrafish hearts and 3% agarose gel electrophoresis. Arrows indicated the positions of PCR products from the polyadenylated and oligoadenylated RNAs, respectively. (**E**) Quantification of poly(A) proportions of *nd1, cytb*, *cox1* and *cox3* transcripts in the WT and *mto1*^−/−^ zebrafish. (**F**) Poly(A) tail lengths from individually sequenced clones after 3’ end RACE analysis of *nd1* transcripts in the *mto1*^−/−^ and WT zebrafish hearts. Graph details and symbols are explained in the legend to Figure 2.

To test whether MTO1 directly interacts with MTPAP, we carried out the immunoprecipitation assay using MTPAP and FLAG antibodies in HEK293T cell lines overexpressed with human FLAG-tagged MTO1. As shown in Figure 8A, B, the MTO1-FLAG antibody reciprocally immunoprecipitated to MTPAP, but did not bind to TOM20. These data suggested the potential interaction of MTO1 with MTPAP.

To further investigate the potential interaction of MTO1 with MTPAP, we measured the levels of Mtpap in the *mto1*^−/−^ and WT zebrafish using the western blot analysis. As shown in Figure 8C, the levels of Mtpap in *mto1*^−/−^ zebrafish were 30%, relative to the mean values measured in WT zebrafish. The potential direct interaction suggested that the deletion of *mto1* affected the polyadenylation of mitochondrial mRNAs. To test this hypothesis, we performed *in vitro* polyadenylation experiments by analyzing the 3′ ends of mitochondrial *nd1, cox1*, *cox3*, *cytb* transcripts in *mto1*^−/−^ and WT zebrafish. Frist, we analyzed the poly(A) tail levels of *nd1*, *cytb*, *cox1* and *cox3* transcripts by 3′RACE, followed by using 3% agarose gel electrophoresis. As shown in Figure 8D, the poly(A) tails of *nd1, cox1* and *cox3* but not *cytb* transcripts were significantly decreased in *mto1*^−/−^ fish line, as compared with those in WT fishes. In particular, the poly(A) levels of *cox1*, *cox3*, *nd1*, *cytb* in *mto1*^−/−^ mutant zebrafish were 18%, 25%, 71% and 90%, relative to those of WT zebrafish, respectively (Figure 8E). We then examined the poly(A) tail length of *nd1* transcripts by 3′RACE, followed by cloning and sequencing in *mto1*^−/−^ and WT zebrafish. As shown in Figure 8F, the poly(A) tail length of *nd1* in the *mto1*^−/−^ zebrafish was about 33 nt, while the average poly(A) tail length of *nd1* in the WT zebrafish was about 47 nt. These data indicated that the ablation of Mto1 altered the polyadenylation of mitochondrial mRNAs.

## DISCUSSION

The pathogenic mechanism underlying the mitochondrial transcript maturation deficiency leading to tissue-specific manifestations remains largely elusive. Using *mto1* knock-out zebrafish generated with CRISPR/Cas9 system, we demonstrated the profound impacts of Mto1 deficiency on mitochondrial transcript maturation contributing to the pathogenesis of hypertrophic cardiomyopathy. In particular, the deletion of *Mto1* led to the pleiotropic effects on the posttranscriptional nucleotide modification, aminoacylation and stability of mitochondrial tRNA as well as the polyadenylation of mRNA. In fact, Mto1 is a highly conserved tRNA modifying enzyme responsible for the biogenesis of τm^5^U at the wobble position of mitochondrial tRNA^Glu^, tRNA^Gln^, tRNA^Lys^, tRNA^Trp^ and tRNA^Leu(UUR)^ (30,32,33,36). Therefore, the Mto1 deficiency affected both structure and function of mitochondrial tRNAs. In this study, we demonstrated that the MTO1-deficiency perturbed the tertiary structures and stability of tRNA^Gln^, tRNA^Lys^, tRNA^Trp^ and tRNA^Leu(UUR)^ containing the thiocarbonyl group, but not tRNA^Ala^ lacking this modification. Especially, the instability of these tRNA were evidenced by various electrophoretic mobility changes and sensitivity to S1-mediated digestion of these tRNAs in the *mot1*^−/−^ zebrafish, as in the case of *gtpbp3*^ko^ zebrafish (56). The previous investigations showed that the MTO1-deficiency resulted in the defect in τm^5^U synthesis in both human and mice cells (36,39,43). Due to the facts that the thiolation at position 2 was independent of the modification at position 5 (27,74), *mto1^−^^/^*^*−*^ zebrafish exhibited similar biochemical defects in tRNA metabolisms to those in *gtpbp3^−^^/^*^*−*^ zebrafish (56), but differed from those in *trmu^−^^/^*^*−*^ zebrafish (53). These included no effects of *Mto1* ablation on the 2-thiouridylated levels at U34 of tRNA^Lys^, tRNA^Glu^ and steady-state levels of tRNA^Glu^, tRNA^Gln^, tRNA^Lys^, tRNA^Trp^ and tRNA^Leu(UUR)^, in contrast with the deficient 2-thiouridylation at U34 of tRNA^Lys^, tRNA^Glu^ and tRNA^Gln^ and global decreases of tRNAs in the *trmu*^KO^ zebrafish and human cells bearing the *TRMU* mutations (30,31,53). However, the *mto1^−^^/−^* zebrafish exhibited reduced aminoacylation efficiencies of tRNA^Lys^, tRNA^Gln^, tRNA^Trp^ and tRNA^Leu(UUR)^ with the taurine modification but no changes of those tRNAs without the taurine modification, in contrast with the increasing efficiencies of tRNA aminoacylations in the *gtpbp3*^KO^ zebrafish (56). These discrepancies may be attributed to the different types of modifications at U34 in mitochondrial tRNA^Glu^, tRNA^Gln^, tRNA^Lys^, tRNA^Trp^ and tRNA^Leu(UUR)^, even though these tRNAs shared identical taurine modification at U34 (26,27). These data highlighted the central roles of Mto1 in the mitochondrial tRNA maturations.

Mitochondrial tRNA nucleotide modifications are emerging as a key regulator of mitochondrial gene expression (75). Strikingly, we demonstrated that the ablated *mto1* impaired the polyadenylation of *cox1*, *cox3*, and *nd1* mRNAs in the zebrafish. The polyadenylation on the 3′ ends of animal mitochondrial mRNAs was synthesized by own MTPAP (14–16,76). In fact, LRPPRC (mitochondrial mRNA stabilising factor), SUV3 (ATP-dependent RNA helicase) and PNPase (polynucleotide phosphorylase) regulates the polyadenylation of mitochondrial transcripts by modulating the function of MTPAP (68,76–77). In the study, the RNA-seq approach, together with real time quantitative PCR, showed the marked decreases in the expression levels of *mtpap* in the *mto1*^KO^ zebrafish heart, as compared with those in WT zebrafish. These data implicated the potential interaction of Mto1 with Mtpap to mediate the polyadenylation of mitochondrial mRNAs. The specific interaction of MTO1 with MTPAP was confirmed by immunoprecipitation assays. The potential interaction was further supported by the marked reductions in the levels of Mtpap protein in the *mto1*^−/−^ zebrafish, as compared with WT zebrafish. These deficiencies manifested significant decreases in the levels of poly(A) tails in the *nd1*, *cox1* and *cox3* mRNAs and shortening lengthes of poly(A) tail in *nd1* mRNA. The defective adenylation of mRNA may affect the stability of these mitochondrial transcripts. These results were comparable with the alterations in polyadenylation of mitochondrial transcripts observed in the human cell lines bearing the *MTPAP* mutations linked to spastic ataxia (14–16,78,79). These findings indicated the critical role of *mto1* in the posttranscriptional regulation of mitochondrial mRNAs.

The pleiotropic effects of Mto1 deficiency on tRNA and mRNA maturations impaired the mitochondrial protein synthesis, as in the case of human cell lines harboring the *MTPAP* mutations (14). In the present study, the marked decreases in the levels of Nd1, Co2 and Atp8 and Tufm were observed in the *mto1^−^^/^^−^* zebrafish, while the levels of Sdha, Atp5a and Tfam did not differ significantly between *mto1^−^^/^^−^* and WT zebrafish. These results were in contrast with the observations that *gtpbp3^−^^/^^−^* zebrafish exhibited the various reductions in the levels of Atp5c, Sdha and Tfam (56). These data indicated the pronounced effects of *mto1* on mitochondrial translation. The *mto1* mutation-induced translational defects may yield a buildup of unfolded and/or unassembled subunits of OXPHOS, thereby altering the activities of OXPHOS complexes and subsequent failure of cellular energetic process (80). In this study, we showed that *mto1* deletion in zebrafish resulted in significant decreases in the steady state levels and activities of complex I and IV. These deficiencies of complex I and complex IV were in a good agreement with the defects in the complex I and complex IV in the human cells bearing the *MTO1* mutations and *Mto1*^KO^ mice (36,39,43,44). These mitochondrial dysfunctions were further verified by the reduced activities of COX and increased fragmented mitochondria in the cardiomyocytes of *mto1*^−/−^ mutant zebrafish. Notably, human cell lines carrying the MTPAP p.N478D mutation exhibited defects in the complexes I and IV (14).

The *mto1* and *mtpap* were highly expressed in the heart of 2 dpf zebrafish, but decreased at 3 dpf, implicating their critical roles in the heart function. These suggested that mitochondrial dysfunction caused by Mto1-deficieny altered the heart development and function. In this study, the *mto1^−^^/^^−^* mutant larvae at 2 dpf revealed the perturbed S-loops processing in heart. The cardiac defects were further evidenced by the fact that higher percentage of *mto1^−^^/^^−^* mutant zebrafish had no-loop heart. These results indicated that the loss of Mto1 affected the embryonic heart development. Notably, *mto1^−^^/^^−^* adult zebrafish exhibited the hypertrophy of cardiac myocytes and myocardial fiber disarray. The abnormal myocytes included the enlarged size, bizarre-shaped nuclei and disorganized patterns. Furthermore, *mto1*^−/−^ zebrafish displayed defects in cardiac myofibrils and widened I-bands. These data demonstrated that *mto1^−^^/−^* zebrafish recapitulated the clinical phenotypes in HCM patients carrying the *MTO1* mutations (43–46). Therefore, our findings may provide new insights into the pathophysiology of hypertrophic cardiomyopathy, which was manifested by defective maturations of mitochondrial transcripts.

## DATA AVAILABILITY

The authors declare that [the/all other] data supporting the findings of this study are available within the article [and its supplementary information files].

## Supplementary Material

gkab228_Supplemental_File
