# Supplementary material for: Ablation of Mto1 in zebrafish exhibited hypertrophic cardiomyopathy manifested by mitochondrion RNA maturation deficiency
Source: Nucleic Acids Res. 2021 Apr 9;49(8):4689–704. doi: 10.1093/nar/gkab228 (PMC8096277; doi:10.1093/nar/gkab228)
Supplement: gkab228_Supplemental_File [file gkab228_supplemental_file.pdf]

Supplemental Data

Figure S1

Figure S2

Figure S3

Figure S4

Figure S5

Table S1

Table S2

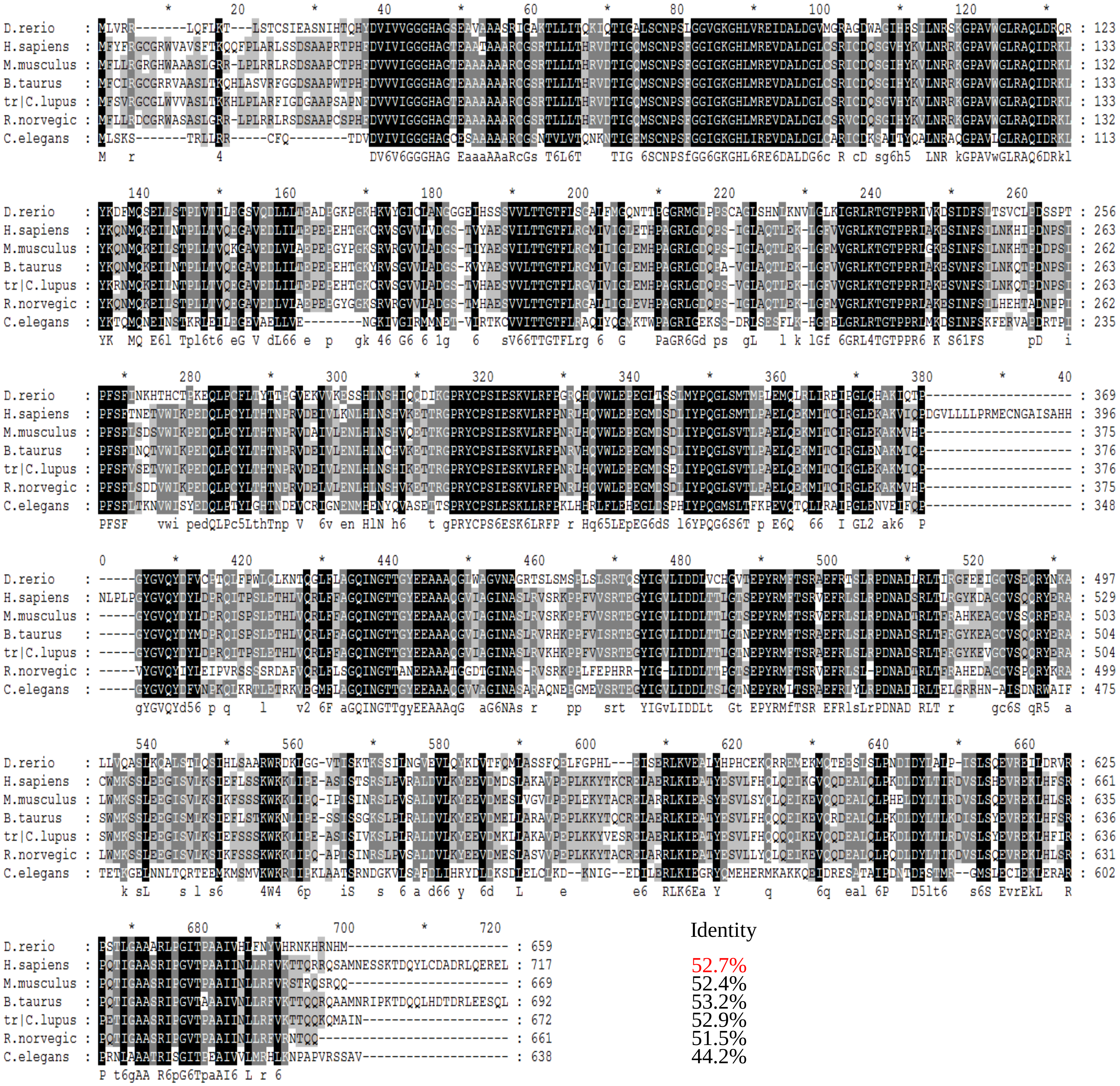

Supplemental Figure S1: Amino acid sequence multiple alignment of the MTO1 family encompassing *D.rerio*, *H.sapiens*, *M.musculus*, *R.Norvegicus*, *C.lupus*, *B.taurus*, and *C.elegans* performed using ClustalW. zebrafish Mto1 shares about 52.7% sequence identity with human MTO1 .(*Danio rerio*: NP_001076478.1; *Homo sapiens*: NP_598400.1; *Mus musculus*: NP_080934.2; *Rattus norvegicus*: NP_001100311.1; *Canislupus*: XP_532202.4; *BosTaurus*: XP_005210702.1; *Caenorhabditis elegans*: NP_496169.1)

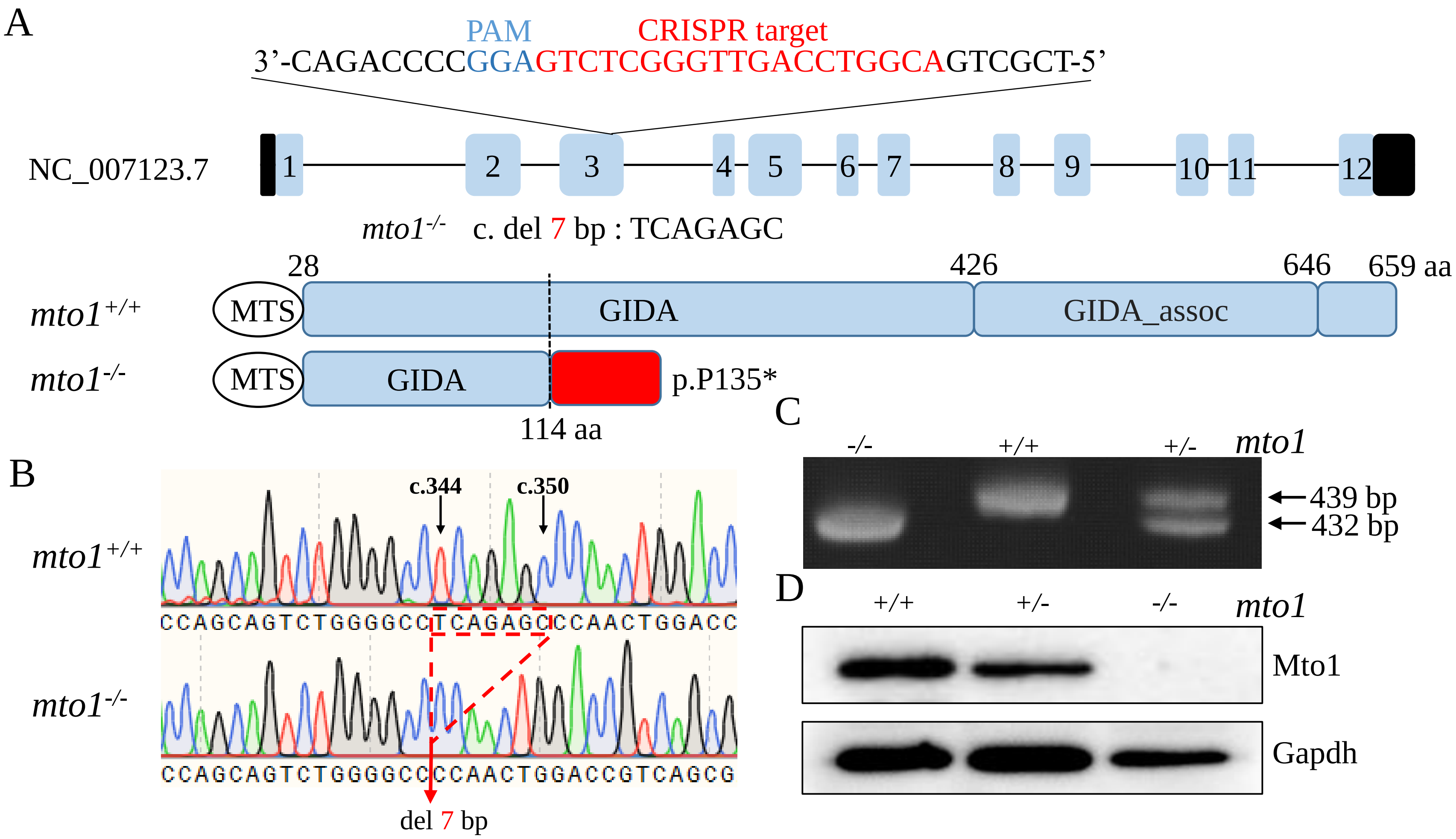

supplemental Figure S2 (A) Schematic representation of CRISPR/Cas9 target site at exon 3 of zebrafish *mto1* gene as used in this study. Light blue boxes indicate coding region, black boxes indicate untranslated regions of exons and lines between exons indicate introns. The resultant truncated 135 aa non-functional protein caused by frame shifting deletion in *mto1* is shown in diagram. (B) Partial sequence chromatograms of exon 3 in the *mto1* gene in the *mto1*$^{+/+}$ and *mto1*$^{-/-}$ zebrafish. The red arrow indicates the location of the nucleotide changes. (C, D) Genotyping of *mto1*^{del7bp} by the PAGE and Western blot analyses.

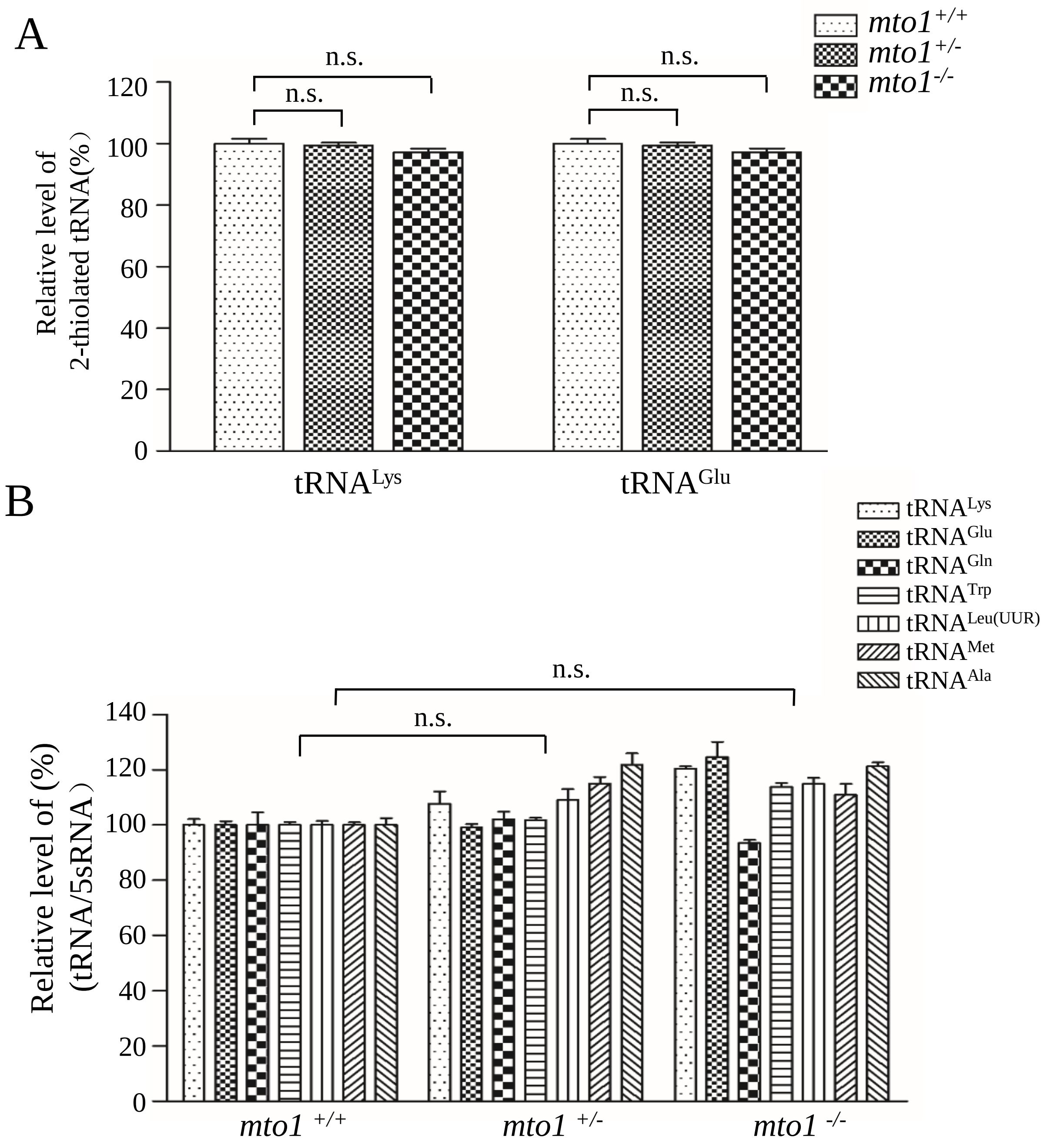

Supplementary Figure S3. Quantification of the levels of mitochondrial tRNAs. (A) Proportion in vivo of the 2-thiolated tRNA levels. The proportion values for the mutant zebrafish are expressed as percentages of the average values for the WT zebrafish. The calculations were based on three independent determinations of each tRNA in each fish. The error bars indicate standard deviations of the means; *P* indicates the significance, according to Student's t test, of the difference between mutant and WT for each tRNA. (B) Quantification of the levels of tRNAs. Average relative levels tRNA content were normalized to the average content in the mutant and WT 5S rRNA, respectively. The values for the *mto1*$^{+/-}$ and *mto1*$^{-/-}$ zebrafish are expressed as percentages of the average values for the WT zebrafish. The calculations were based on three independent determinations. Graph details and symbols are explained in the legend to Figure 3.

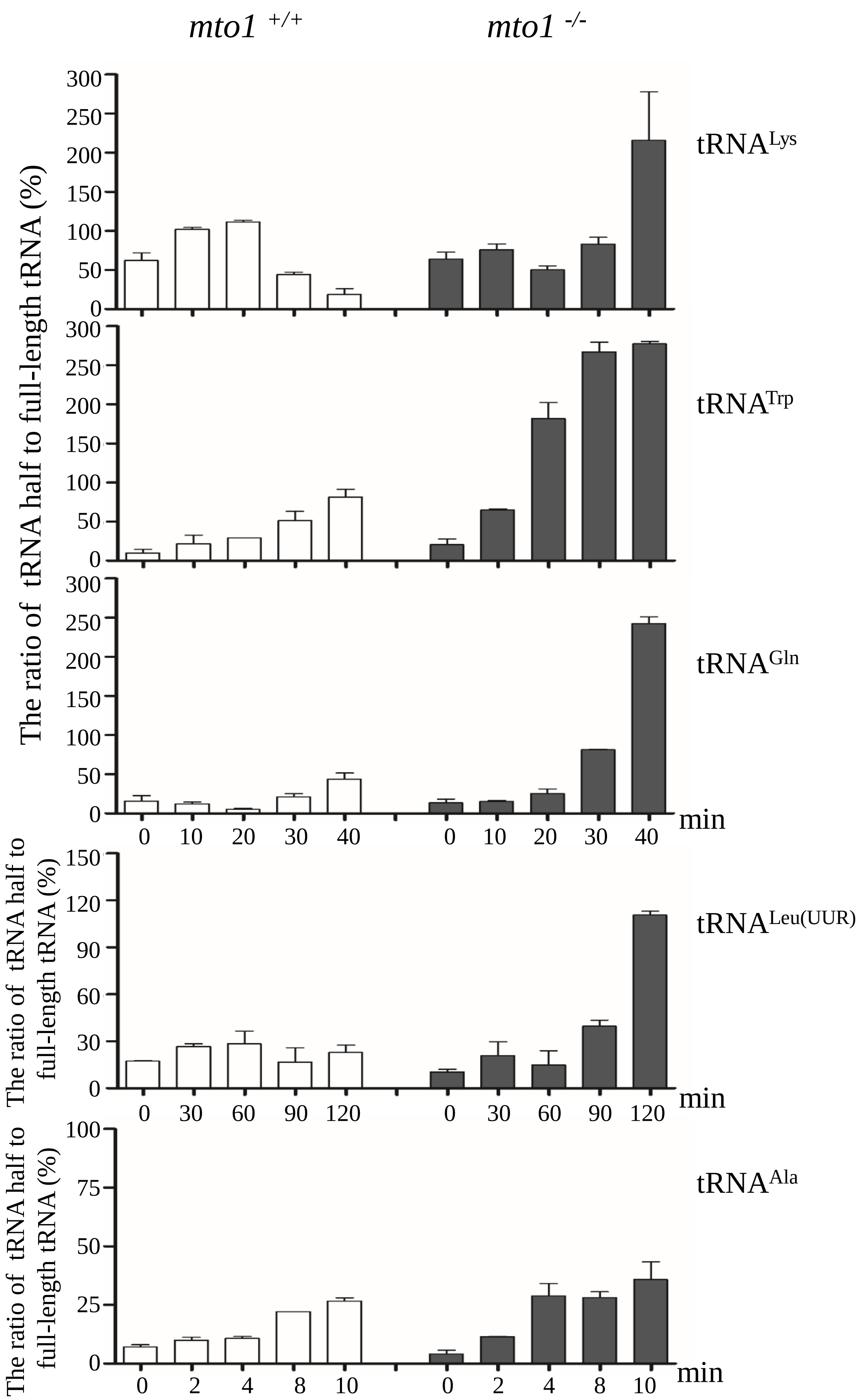

Supplementary Figure S4. The ratios of tRNA half to full-length tRNA. The ratios of tRNA half to full-length tRNA with S1 digestions of $tRNA^{Lys}$, $tRNA^{Trp}$, $tRNA^{Gln}$, $tRNA^{Leu(UUR)}$ and $tRNA^{Ala}$, purified from *mto1*$^{-/-}$ and WT zebrafish. The calculations were based on three independent determinations.

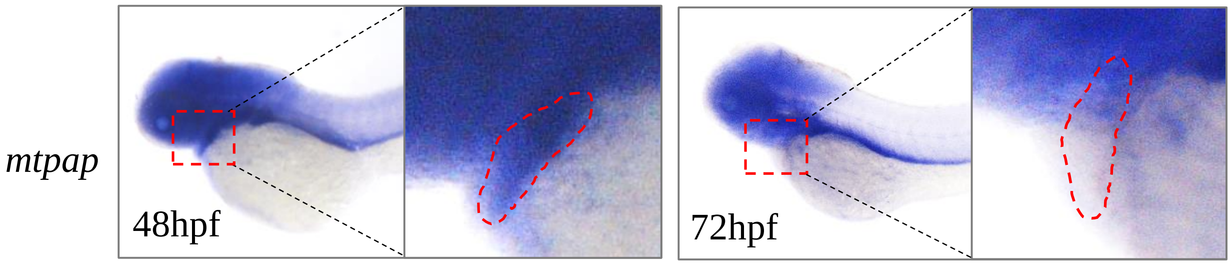

Supplementary Figure S5. The expression patterns of Zebrafish Mtpap. Whole-mount in situ hybridization (WISH) analysis of *mtpap* on WT larval zebrafish at 48 hpf and 72 hpf. Insets show higher magnifications of heart.

Supplementary table S1. Sequences of oligodeoxynucleosides for Whole-mount *in situ* hybridization, Quantitative Real-time PCR, Immunoprecipitation, Poly(A) tail-length.

Experiment	Oligo	Sequence
ISH probe	Mto1-Probe-F	CCAGACTATTGGGGCACT
	Mto1-Probe-R	TTGACAATACGGGGAGGA
	Mtpap-Probe-F	AAGCAACTATCCCACGCCAA
	Mtpap-Probe-R	GAAGGCTGATGAGCAAAGCG
	Cmlc2-Probe-F	CAGACCAACAGCAAAGCAG
	Cmlc2-Probe-R	TGCAACTGAGTATGAAGTTTA
Quantitative Real-time PCR	qpcr-aco2-F	ATTGAGCGAGATGGCTATGC
	qpcr-aco2-R	CAAAAGCATGAGTTGCAGGG
	qpcr-ep300a-F	CACCTCTTAACCAGGGAAGC
	qpcr-ep300a-R	TAACGGCATTTGTCCCTGTT
	qpcr-mtpap-F	ACTGTAAACAGCTTCGGCAA
	qpcr-mtpap-R	CACCTGGTACTCCAGAGACA
	qpcr-pnpo-F	CTTGCAACAGCGACTAAGGA
	qpcr-pnpo-R	CTCCAGCTCTGAACCTTTCC
	qpcr-tnrc6b-F	GGCATGGGCTTAAACGAGTA
	qpcr-tnrc6b-R	CATCCCTCCTCCGCTACTAA
	qpcr-lpl-F	GAATACACGGCGAGAAGGAG
	qpcr-lpl-R	GTCAACAGGAAAGACACGGT
	qpcr-pgp-F	TGTGTCGCAAGTCAGTTCAA
	qpcr-pgp-R	TGCGGACATTCACTCTTCTG
	qpcr-mrtfab-F	GCCATTGAAGGAACGCAAAA
	qpcr-mrtfab-R	TTCCTCTGTTCGTGGAAAGC
	qpcr-ef1α-F	TACAAATGCGGTGGAATCGAC
	qpcr-ef1α-R	GTCAGCCTGAGAAGTACCAGT
	qpcr-mto1-F	GCTGGGCAAATAAATGG
	qpcr-mto1-R	GTGCGTGACAAGGACAAT
Immunoprecipitation (IP) assay	MTO1 flag XhoI R	CCTCGAGGCATAACTCTCTCTCTTGAAGTCTGT
	MTO1 flag EcoRI F	GGAATTCCGCCACCATGTTCTACTTCCGAGGCT
Poly(A) tail-length assay	linker	Phospho-ATGTGAGATCATGCACAGTCATA-NH2
	anti-linker	TATGACTGTGCATGATCTCACAT
	inner anti-linker	GACTGTGCATGATCTCACAT
	cox1 upper	CTACCCAGACGCCTATGCAC
	cox1 lower	ACCGCAACAAATGTAGAATG
	cox3 upper	GCCTCATGGAAGGAGAACGA
	cox3 lower	AGCTGTATGCCTTCTACGCC
	nd1 upper	AGCATCGTTTACCCCAGATGC
	nd1 lower	CCCACGATTCCGATACGACC
	cytb upper	TCGCATACGCCATTCTACGA
	cytb lower	ACCCCTACATCATCATTGGACA

Supplementary Table S2. The sequence of tRNA probe

tRNA Probe	Sequence
mt-tRNA^{Lys}	TCACTAAGGGTGGTCGGTAAGCACCAAGTT
mt-tRNA^{Glu}	AATTCTTACTCAGACTTTAACTGAGACCGG
mt-tRNA^{Gln}	TTAGAAAGAAGGGGGTCGAACCCATGCCCA
mt-tRNA$^{Leu（UUR）}$	GGCCTTTTGCAATTACCGAGCTCTGCCATC
mt-tRNA^{Trp}	TCTACTGAGAGCTTTGAAGGCTCTTGGTCT
mt-tRNA^{Ala}	TAGGACTTACAGACGTTACTCCGCATCTTC
mt-tRNA^{Met}	TGATGAAGGAGGGACTTTAACCATCATGTT
5S rRNA	GCAACCTAGTTTTCCCATGTGGTCTCCCAT
